# Photoinduced Immobilization on Two-Dimensional Nano Borophene Spatially Orients Capture Antibody for Highly Sensitive Biological Interactions

**DOI:** 10.1021/acscentsci.5c00474

**Published:** 2025-07-17

**Authors:** Satheesh Natarajan, Ketan Dighe, Teresa Aditya, Pranay Saha, David Skrodzki, Purva Gupta, Nivetha Gunaseelan, Shraddha Krishnakumar, Dipanjan Pan

**Affiliations:** a Department of Nuclear Engineering, 8082 The Pennsylvania State University, University Park, Pennsylvania 16802, United States; b Department of Biomedical Engineering, The Pennsylvania State University, University Park, Pennsylvania 16802, United States; c Department of Materials Science and Engineering, The Pennsylvania State University, University Park, Pennsylvania 16802, United States; d Huck Institutes of the Life Sciences, University Park, Pennsylvania 16802, United States

## Abstract

Two-dimensional (2D) nanomaterials are of great interest due to their unique properties and broad biological applications. Among these, borophene, a single-atom-thick boron sheet with a honeycomb structure, exhibits exceptional structural, electronic, and mechanical characteristics, making it a promising candidate for sensing, electronics, and biosensing. In this study, we report on a liquid-phase exfoliation method to synthesize stable borophene nanosheets and introduce a photoinduced immobilization technique to functionalize their surfaces with antibodies. By exploiting borophene’s electron-deficient nature, we enable strong covalent bonding with electron-rich thiol groups in antibodies. UV irradiation cleaves antibody disulfide bonds, generating free thiols that form stable boron–sulfur bonds with borophene, resulting in spatially oriented antibodies that preserve antigen-binding activity. We demonstrate the application of these functionalized nanosheets in a lateral flow immunoassay (LFIA), a key tool in point-of-care diagnostics that is often limited by poor antibody orientation. The developed LFIA detects HMGB-1, a potential endometriosis biomarker, in menstrual effluent with results in 10 min and a limit of detection of 40 pg/mL. This performance surpasses that of conventional LFIAs, showing high sensitivity, specificity, and no cross-reactivity with common blood proteins. This study highlights a novel, reagent-free strategy for functionalizing borophene, enhancing its potential in biosensing applications.

## Graphical abstract

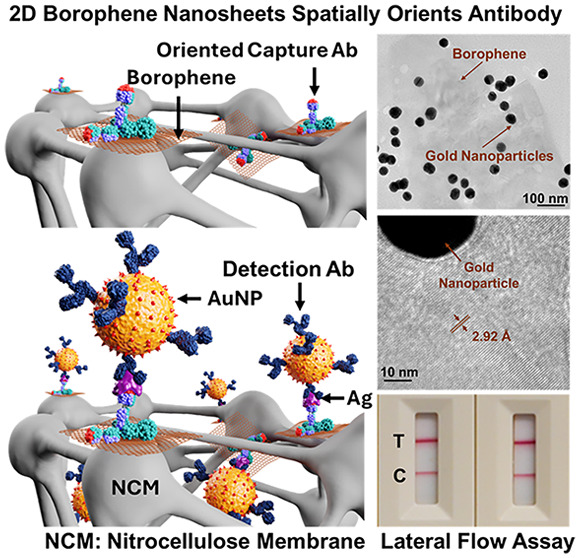

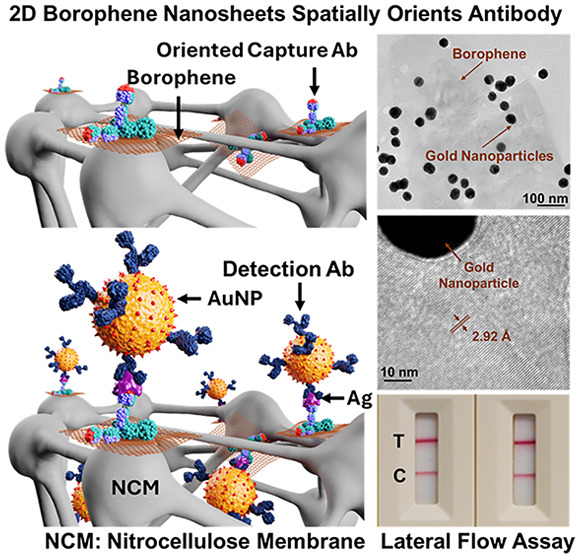

## Introduction

Over the past two decades, the discovery of two-dimensional (2D) nanomaterials with atomic-scale thickness has transformed fundamental nanoscience and nanotechnology. The inception of this discipline pertains to the pioneering synthesis of graphene, which paved the foundation for further exploration of various 2D nanomaterials.^1,2^ Since then, many analogous materials such as hexagonal boron nitride (h-BN) and graphitic carbon nitride (g-C_3_N_4_) have garnered significant attention due to their exceptional mechanical strength, superior electrical mobility, and distinctive thermal properties.^3,4^ These unique properties have facilitated a wide array of applications, spanning electronics, energy storage, and biosensing.^5^ In the area of sensing, graphene-based sensors have remarkable versatility in detecting analytes, encompassing environmental pollutants such as heavy metals and pesticides, as well as biological markers like enzymes, proteins, and nucleic acids.^6,7^ Their inherent sensitivity and versatility facilitate their application in wearable sensors for mechanical strain and real-time physiological parameter monitoring. On the other hand, challenges such as graphene’s intrinsic lack of an electronic bandgap and certain performance limitations have prompted researchers to investigate alternative 2D materials beyond graphene, including silicene, germanene, borophene, transition-metal dichalcogenides (TMDs), MXenes, and metal–organic frameworks (MOFs).^8,9^

Among these, borophene, a recently synthesized monoelemental member of the Xene family and a two-dimensional (2D) allotrope of boron, has garnered significant attention due to its unique structural, electronic, optical, and physicochemical properties, as well as its complex allotropic forms, which distinguish it from other 2D materials.^10,11^ Notably, both theoretical and experimental studies have demonstrated borophene’s outstanding thermal conductivity, mechanical strength, and tunable electronic characteristics, positioning it as a versatile platform for a wide range of applications^12,8,13,14^ Its cohesive atomic structure provides remarkable mechanical durability, while its high carrier mobility, tunable band gap, exceptional electrical conductivity, and intrinsic superconducting behavior collectively facilitate efficient charge transport and broaden its potential for next-generation nanoscale devices.^15–18^ Recent studies have highlighted borophene’s outstanding performance in high-performance energy storage, gas sensing, and, importantly, advanced electronic and optoelectronic applications.^16,17,19,18^ Moreover, experimental studies have validated borophene’s promise in these fields, demonstrating its superior electronic conductivity, optical response, and catalytic activity compared to conventional materials. Furthermore, borophene has emerged as a biocompatible and biodegradable material, demonstrating low cytotoxicity in vitro and minimal adverse effects in vivo, making it highly suitable for biomedical applications.^15,20,21^ Environmentally, its piezocatalytic activity facilitates the efficient degradation of organic pollutants, highlighting its potential in sustainable environmental technologies.^21,22^

Comprehensive reviews further highlight borophene’s broad applicability and its rapidly evolving role in addressing current challenges in materials science, energy conversion, nanoelectronics, biomedicine, and environmental remediation. Collectively, these advances establish borophene as an exceptionally promising 2D nanomaterial, capable of overcoming existing limitations in multifunctional device development and fostering transformative innovations across both academic and industrial domains.^12,17–19,23^

Before the practical implementation of any application, the successful and efficient synthesis of high-quality materials constitutes a critical experimental step. Although numerous theoretical conformations of borophene synthesis on various metal substrates have been proposed, only a limited number of experimental procedures have been reported thus far.^11,24^ A few studies have demonstrated the synthesis of atomically thin crystalline boron films on metal surfaces through vacuum deposition methods, such as molecular beam epitaxy (MBE) and chemical vapor deposition (CVD).^25^ However, these approaches present several drawbacks, including the necessity for ultrahigh vacuum and low-pressure growth, an additional transfer step that inevitably degrades sheet quality, and inadequate yields, which collectively constrain their practical applications.^26^ As an alternative, liquid-phase exfoliation methods have gained considerable attention for synthesizing and upscaling 2D nanomaterials (specifically 2D nanosheets), owing to their ability to produce materials of high quality in large quantities. Furthermore, some studies have reported the synthesis of few-layer borophene nanosheets via sonication-assisted liquid-phase exfoliation in high-boiling solvents such as dimethylformamide and isopropyl alcohol.^26,27^ However, these solvents complicate purification and removal because their slow evaporation rates promote aggregation of the 2D nanomaterials, thereby rendering them unsuitable for biological applications. To address these challenges, an alternative strategy involving low-boiling solvents has been introduced. By employing low-boiling solvents such as water, stable dispersions of 2D nanomaterials can be achieved and conveniently utilized for various biological applications.^4,28^ Accordingly, in this study, we utilized Milli-Q water as a low-boiling solvent to obtain stable dispersions of borophene nanosheets from bulk boron powder via liquid-phase exfoliation for biological application. The biological interactions of borophene could be of particular interest not only for developing sensing strategies but also for enhancing sensor performance.

Boron atoms in borophene are electron-deficient, facilitating effective surface functionalization with electron-donating agents.^29^ Moreover, its polymorphic nature enables further tuning of properties via various boron–boron bonding configurations. This functionalization enhances biosensing capabilities by facilitating selective interactions with certain bioanalytes, promoting physical adsorption on the surface. Borophene’s anisotropic character, high electroactive surface area (stemming from its chemical and metallic properties), and high electron mobility may further endow it with enhanced selectivity and sensitivity in biosensor development.^29,30^ Recently, several studies have employed borophene nanosheets in combination with MOFs, metals, and other active organic ligands (e.g., polyaniline and phthalocyanine) to synthesize borophene-based nanocomposites for electrochemical biosensors.^31^ However, none of these approaches utilize pristine borophene nanosheets for direct surface functionalization. Therefore, there remains a critical gap in the literature regarding the development of functionalization strategies for pristine borophene nanosheets, either with or without the need for additional functional groups.

Our group recently demonstrated that pristine borophene nanosheets can be directly functionalized using a site-selective boron–sulfur conjugation strategy with thiol-containing amino acids like cysteine.^32^ This work provided a deeper understanding of developing functionalization strategies for borophene nanosheets without additional surface modifications that can be applied to sensing and catalysis.^32^ Building on this foundational approach, we aimed to study the possibility of conjugating biomolecules, specifically antibodies, onto borophene nanosheets. Although antibodies serve as exceptional receptors due to their specificity, flexibility, and reliability, their moderate long-term stability and the necessity for proper orientation and high surface density pose problems for functionalization. Conventional noncovalent or covalent immobilization techniques may reduce antibody immunoaffinity due to steric hindrance.^33^ Site-specific conjugation methods, such as oxidation of carbohydrate moieties in the Fc region to form Schiff bases, improve attachment efficiency; however, they often lead to undesirable side reactions and reduced antibody activity.^34^ There is a need for an effective immobilization strategy that ensures antibodies are attached site-specifically and in an orientation favorable for antigen binding efficiency, without the use of additional chemical reagents and while maintaining their biological recognition activity.^7,34–36^

The photochemical immobilization technique (PIT) is an established and efficient technique for antibody immobilization onto solid surfaces while ensuring optimal exposure of fragment antigen-binding (Fab) fragments.^34,36–39^ In this study, we investigated the potential of utilizing the PIT method to immobilize directly and spatially orient antibodies onto the borophene surface, a promising yet relatively unexplored approach. The PIT technique utilizes UV irradiation of IgG antibodies to generate four free thiol groups in the Fab region, two of which are available for covalent binding to metal/metallic surfaces. The thiol groups, being electron-rich, can interact with the electron-deficient boron atoms of borophene, resulting in the formation of a boron–sulfur bond. This approach can use pristine 2D borophene nanosheets to spatially orient antibodies, creating a bioactive surface for sensitive biomolecule recognition. To demonstrate the practical application, the developed borophene–antibody conjugates were then used as capture probes to develop a lateral flow immunoassay (LFIA) for women’s health diagnostics. The introduction of a 2D borophene nanosheets, together with a photoactivation strategy, enables the uniform immobilization of antibodies while preserving their antigen-binding efficacy, thereby enhancing the antigen detection performance of the LFIA. To assess the efficiency of these techniques, along with their sensitivity, limit of detection (LOD), and dynamic clinical detection range, we targeted HMGB-1,^40^ a biomarker for endometriosis, using menstrual blood to advance women’s health diagnostics. Despite the significant potential of menstrual effluent^41^ as a diagnostic tool for women’s health, it often faces substantial challenges due to social stigma and limited access to affordable diagnostic methods. These challenges contribute to delays in diagnosing endometriosis, with one study of 218 women revealing an average delay of over 8 years in the United Kingdom and up to 12 years in the United States.^42^ By leveraging the novel 2D material borophene, this approach offers a unique opportunity to reduce menstrual stigma while advancing women’s health.

In a nutshell, we prepared a stable dispersion of χ_3_ borophene nanosheets from bulk boron powder in an appropriate volume of low-boiling solvent (H_2_O) via liquid-phase exfoliation.^32^ We then employed these nanosheets to spatially orient antibodies on their surface using PIT. The resulting nanosheets, bearing spatially oriented antibodies, were subsequently utilized to develop an ultrasensitive LFIA device capable of detecting HMGB-1 in menstrual effluent at low concentrations within minutes, without requiring pretreatment. We believe that the novelty of this approach and the assay’s high sensitivity will attract considerable interest in advancing borophene-based biosensing applications. Moreover, this study highlights the broader research opportunities essential for realizing next-generation biotechnologies, with 2D materials at their core, and positions borophene as a highly promising class of nanomaterial for cutting-edge biosensors and advanced healthcare applications.

## Results and Discussion

### 3D Bulk Boron to 2D Borophene Nanosheets

Borophene nanosheets were synthesized via probe sonication-assisted liquid-phase exfoliation of bulk boron powder in Milli-Q water (Figure 1a). Water was chosen for its low boiling point and compatibility with biological applications.^30,43^ While high-boiling solvents such as dimethylformamide (DMF) or *N*-methylpyrrolidone (NMP) are commonly used to stabilize 2D nanomaterial dispersions by lowering interlayer van der Waals forces, their removal is challenging and can lead to aggregation, limiting their use in biomedical contexts.^44–46^ In contrast, Milli-Q water (18.2 MΩ cm resistivity) minimizes the generation of reactive oxygen species (ROS) and, under probe sonication, generates intense cavitation microenvironments that facilitate the intercalation of water molecules between boron layers, weakening interlayer attractions and promoting exfoliation.^43,47^ Although water is generally less effective than some organic solvents for liquid-phase exfoliation due to its higher polarity and weaker biaxial straining effect,^45^ it can still yield thin borophene sheets, particularly when combined with optimized sonication and postprocessing conditions.^30,43,46^ Controlled sonication time under an inert atmosphere helps suppress oxidation, and subsequent filtration via centrifugation at 3000–4000 rpm allows for the selective isolation of 3–6 layers of borophene nanosheets, consistent with prior studies reporting layer-thickness^46^ control via centrifugation speed. As evidenced by B–OH vibrational features in FTIR in the literature, surface hydroxylation further stabilizes the exfoliated nanosheets in aqueous media and enhances their dispersibility for downstream applications^30,32,48,49^ This approach aligns with recent advances in solvothermal and surfactant-assisted exfoliation, demonstrating that polar solvents and careful process control can yield high-quality borophene nanosheets suitable for biological use. The detailed procedure is described in Materials and Methods.

**Figure 1:**
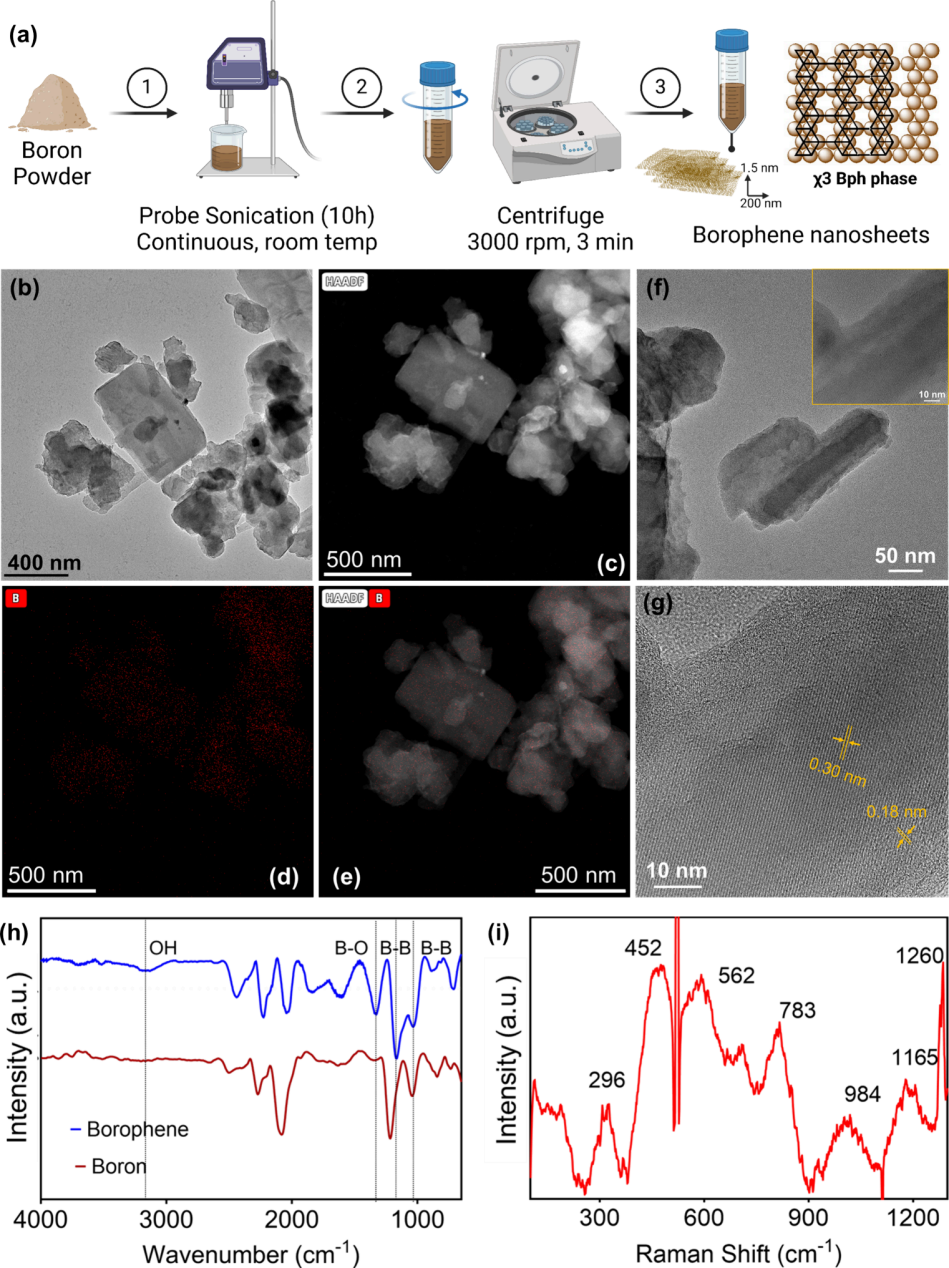
(a) Synthesis and experimental conditions for borophene nanosheets resulting in predominantly χ_3_ phase. Schematic representation of the representative lattice arrangements of χ_3_ borophene phase. Comparative characterization of χ_3_ borophene nanosheets. (b) TEM image and (c) HAADF image with (d) boron EDX map, (e) overlay of HAADF and boron EDX map. (f) HR-TEM image of borophene. The inset shows lattice fringes corresponding to predominantly χ_3_ phase. (g) Calculated lattice fringes using ImageJ software. (h) FT-IR spectra of pristine boron powder and χ_3_ borophene nanosheets in an anhydrous state. (i) Raman spectra of χ_3_ borophene nanosheets in aqueous media.

### Characterization of Borophene Nanosheets

Controlled probe sonication of pristine bulk boron powder at room temperature facilitated the production of free-standing synthesized 2D borophene nanosheets, with an average hydrodynamic diameter of 200 ± 15 nm, as measured by dynamic light scattering (DLS) (Figure S1).^32^ The size of the synthesized nanosheets results from the higher localized temperature in the vicinity of the solvent matrix. The resulting aqueous suspension of borophene was subsequently processed using centrifugation at 3000 rpm followed by filtration to isolate the final nanosheet suspension. Postfiltration, the nanosheets were found to have an average hydrodynamic diameter of 160 ± 20 nm, as determined by DLS. Scanning electron microscopy (SEM) was employed to study and compare the morphological changes between pristine boron powder (bulk boron) and exfoliated borophene nanosheets (Figure S2). SEM images revealed a more flakelike two-dimensional (2D) structure in borophene, whereas pristine boron appeared solid and three-dimensional (3D). The morphology of the nanosheets was further characterized using transmission electron microscopy (TEM), which revealed nanosheet-like structures measuring approximately 200 nm (Figure 1b). High-angle annular dark-field scanning transmission electron microscopy (HAADF-STEM) coupled with energy-dispersive X-ray spectroscopy (EDX) elemental mapping confirmed the presence of boron (Figures 1c–e, S3, and S4). Furthermore, high-resolution transmission electron microscopy (HR-TEM) images revealed lattice fringes of borophene obtained from liquid-phase exfoliation (Figure 1f). The parallel lattice fringes visible throughout the sample indicate a highly ordered crystal structure. The scale bar (10 nm) facilitates measurement of these fringes, which appear to have a moiré interference pattern spacing in the range of 0.29 ± 0.01 and 0.17 ± 0.01 nm (Figure 1g). This lattice spacing is consistent with interatomic distances reported for χ_3_-borophene in prior studies.^30^ Moreover, these spacings differ from those of β_12_ borophene and β-rhombohedral boron.^30,49,50^ The uniform fringe patterns and absence of significant defects or distortions suggest the successful synthesis of high-quality borophene sheets predominantly in the χ_3_ phase. Additionally, FT-IR was performed to confirm the borophene signature bonds in the synthesized nanosheets. The FT-IR spectra (Figure 1h) observed between pristine boron powder and the synthesized borophene nanosheets arise from structural and chemical transformations during liquid-phase exfoliation. In the hydroxyl region (∼3400 cm^–1^), borophene exhibits a pronounced broad band, indicative of substantial surface hydroxylation due to water interactions during aqueous exfoliation and a high tendency for edge oxidation in the reaction medium. In contrast, bulk boron shows minimal hydroxyl signatures, consistent with its lower surface-to-volume ratio and limited edge oxidation. Additionally, in the B–O bonding region (1600–2000 cm^–1^), borophene displays sharper, more intense peaks than bulk boron, reflecting increased oxidation at the edges and surfaces of the nanosheets. This aligns with the exfoliation process, which exposes reactive boron atoms to dissolved oxygen. With its intact 3D covalent network, pristine boron shows weaker B–O vibrations, indicating minimal oxidation. Finally, the B–B bonding region (800–1200 cm^–1^) further distinguishes the two: borophene exhibits sharper and more intense B–B vibrational modes, characteristic of its 2D planar structure. Bulk boron shows broader, less-defined B–B peaks, consistent with its 3D β-rhombohedral configuration.^22,51,52^ We also utilized X-ray photoelectron spectroscopy (XPS) to differentiate between pristine boron powder and synthesized borophene nanosheets. The B1s spectrum of the pristine boron powder indicated only one peak corresponding to B–B (187.33 eV) (Figure S5a). However, the B1s spectrum of borophene reveals three distinct peaks at 187.4, 188.6, and 192.0 eV, corresponding to B–B, B–O, and B_2_O_3_ bonds, respectively (Figure S5b). These peaks represent relative contents of 56% for B–B, 36% for B–O, and 8% for B_2_O_3_. The presence of the B_2_O_3_ peak is likely attributed to electrochemical reactions occurring during the exfoliation process. Notably, borophene exhibits a higher proportion of B–B bonds, which may result from the exposure of freshly cleaved surfaces following exfoliation. Raman spectroscopy was then used to identify the dominant boron phase in the synthesized χ_3_ borophene nanosheets. Based on the synthesis approach, the χ_3_ borophene nanosheets exhibited an isotropic hexagonally bonded phase, defined by a triangular lattice with periodic holes. Raman spectral analysis revealed bands at 296 (A_u_ (Y)), 452 (B_g_^1^ (Y)), 783 (A_g_^3^), 984 (A_g_^2^), and 1165 (A_g_^1^) cm^–1^, confirming the predominance of the χ_3_ phase in the synthesized nanosheets (Figure 1i).^18,48,52,53^ Moreover, the atomic force microscopy (AFM) images of the χ_3_ borophene nanosheets revealed approximately five sheet layers with an overall thickness of 1.10 ± 0.60 nm (Figure S6). The interlayer distance between two adjacent sheets was estimated to be approximately 0.2–0.3 nm. Therefore, a few-layered borophene nanosheets were synthesized using low-temperature liquid-phase exfoliation in an aqueous solution, sonication-assisted intercalation, and bulk boron exfoliation. The method likely involves the dispersion of bulk boron particles into a liquid medium, where the intercalation properties of solvent molecules facilitate a reduction in exfoliation energy, enabling reconstruction and the formation of energy-favorable structures.^2,54^

### Functionalization of Borophene Nanosheets with IgG Antibodies

Following the successful synthesis, characterization, and confirmation of the desired borophene nanosheets, we proceeded to explore their functionalization with antibodies to develop a diagnostic assay. Antibodies (Abs) are the preferred choice for bioreceptors in diagnostic assay development due to their inherent specificity, adaptability, and reliability. However, challenges persist in achieving robust and efficient antibody surface functionalization, primarily due to their moderate long-term stability and the critical requirement to immobilize them with proper orientation and high surface density. The direct introduction of antibodies onto 2D borophene nanosheets presents additional challenges due to the material’s inherent hydrophobicity, lack of reactive sites, and steric hindrance.^55^

To overcome these limitations, we employed a well-established photoinduced immobilization technique, which facilitates the generation of reactive thiol groups in antibodies without compromising their antigen-binding activity. PIT offers significant advantages over traditional methods, including simplicity, speed, and effectiveness enabling the efficient tethering of antibodies onto surfaces with high affinity for thiol groups. The PIT strategy involves the UV irradiation of immunoglobulin G (IgG) antibodies, leading to selective photoreduction that cleaves disulfide bridges within cysteine–cysteine/tryptophan (Cys–Cys/Trp) triads (Figure S7). This process generates four free thiol groups in the Fab fragments, of which two are available for covalent binding to metal surfaces (Figure S7).^38,56^ Moreover, studies have reported that the optimal conditions for PIT with IgG antibodies include an irradiation time of 30 s and an antibody concentration of 50 μg/mL. Under these conditions, the disulfide bridges remain open for approximately 300 s, providing sufficient time for the activated antibodies to attach to the metal surface. Furthermore, it has been reported that immobilization of antibodies via these thiol groups promotes a side-on orientation, in which one Fab domain is bound to the surface, while the other Fab domain adopts orientations within a range of 10° to 90°. This configuration ensures effective exposure of the Fab domain to the analyte in the surrounding medium, thereby enhancing binding efficiency and preserving functionality.^38,39^ Previous research from our group has highlighted the critical role of thiol groups, particularly those from cysteine, an amino acid, in facilitating site-selective boron-sulfur conjugation. This approach leads to the formation of strong covalent bonds with boron atoms, enabling the stable and selective attachment of biomolecules, including antibodies.^32^

Based on these findings, we utilized PIT for the selective photochemical reduction of disulfide bonds in immunoglobulins via UV activation of near-aromatic amino acids using a Trylight lamp. We picked anti-Human HMGB-1 IgG antibodies as a model system since the PIT approach is especially successful for all IgG forms. A standard 10 mm quartz cuvette containing 500 μL solution of anti-Human HMGB-1 IgG antibody at a concentration of 50 μg/mL was housed inside the low-pressure mercury U-shaped UV lamps and irradiated for 30 s at ambient temperature (Figure 2a). Considering the cuvette’s proximity to the lamps and the wrapping geometry, we estimated that the solution received UV irradiation of 0.3 W/cm^2^. The energy of UV photons thus released by the 6 W mercury UV lamp was absorbed by tryptophan residues and subsequently transmitted to surrounding electrophilic species, including adjacent Cys–Cys disulfide bridges. This process resulted in the cleavage of the disulfide bonds and the formation of new reduced thiol (SH) groups. The resulting irradiated antibody solution (100 μL) was then combined with borophene nanosheets (100 μL) at a concentration of 0.175 mg/mL at room temperature, facilitating the functionalization of borophene nanosheets via the formation of boron-sulfur covalent bonds (Figure 2b).

**Figure 2:**
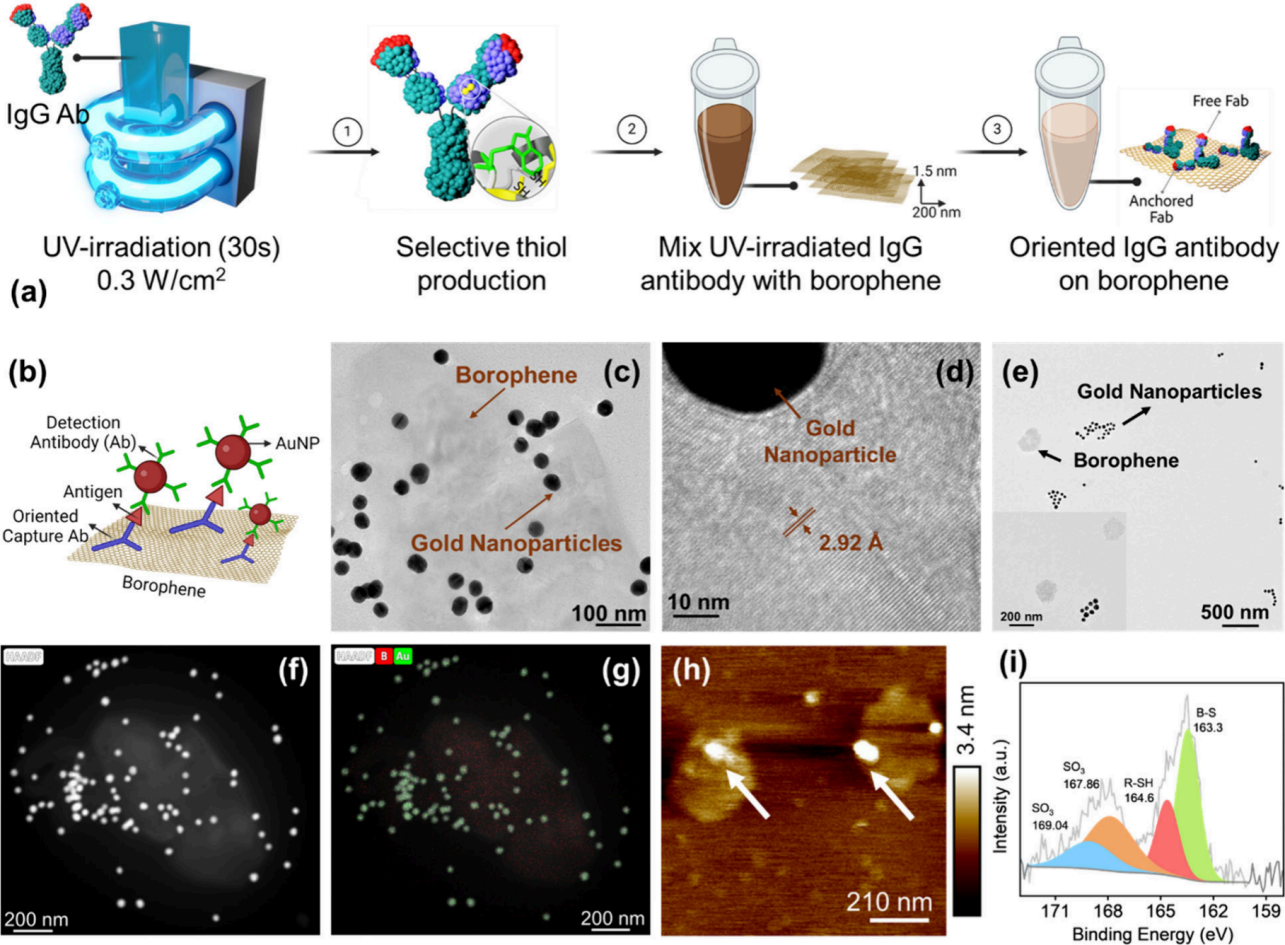
(a) Schematic representation of biofunctionalization of borophene nanosheets with anti-Human HMGB-1 IgG antibodies through PIT. Low-pressure mercury U-shaped UV lamps from Trylight were used to carry out the process. The UV-treated antibody produces four thiol groups (with only two visible in the image). Thiol groups in one Fab region facilitate the immobilization of the antibody to borophene (B–S covalent bond), while the other Fab region remains exposed to the environment for antigen binding. (b) Graphical depiction of the developed immunoassay utilizing the functionalized borophene nanosheets. HMGB-1 antigen and antibodies were used as a model to validate the PIT-based biofunctionalization of borophene nanosheets (c) TEM image illustrating the sandwich formation over borophene nanosheets. Oriented IgG antibodies anchored onto borophene nanosheets form a sandwich complex through interactions between the Fab regions, HMGB-1 antigens, and gold nanoparticles labeled with detection antibodies. (d) High-resolution TEM image showing the lattice fringes in borophene nanosheets. (e) TEM image of the control sample (UV-untreated antibodies over borophene nanosheets). Gold nanoparticles appear separately, agglomerated, and not over borophene sheets. The arrows indicate borophene nanosheets. The inset shows an image at higher magnification. (f, g) HAADF images and EDX mappings of the borophene nanosheet sandwich complex showing the presence of (g) boron (B) and gold (Au). (h) AFM image showing the formation of a sandwich on top of the borophene nanosheet, indicated via an arrow. (i) S 2p XPS spectra of the borophene nanosheets conjugated with UV-treated anti-human HMGB-1 IgG antibodies. The B–S bond is seen at 163.3 eV.

### Quantifying Antibody Functionalization on the Borophene Nanosheet Surface

Following the successful preparation of antibody-functionalized borophene nanosheets via UV-induced thiol generation and subsequent B–S bond formation, we proceeded to characterize the conjugation efficiency and binding mechanism through quantitative analytical techniques. To evaluate and quantify the conjugation efficiency of UV-treated anti-human HMGB-1 IgG antibodies on borophene nanosheets, a Bradford assay using Coomassie Brilliant Blue G-250 dye was conducted (Figure S8a). A linear standard curve with correlation (*R* ^2^ = 0.992) was established using IgG concentrations ranging from 1 to 100 μg/mL (Figure S8b). Following incubation of UV-irradiated antibodies (50 μg/mL) with borophene nanosheets (0.175 mg/mL), a conjugation efficiency of 48% was calculated based on the residual unbound antibody measured in the supernatant. According to the calibration curve, 25.96 μg/mL of free IgG remained, indicating that 24.04 μg/mL IgG (equivalent to 192.3 μg of IgG per mg of borophene) was successfully immobilized on the nanosheet surface. Control experiments using nonirradiated antibodies exhibited negligible binding (<5%), verifying that UV-induced thiol generation is essential for covalent conjugation, presumably via B–S bond formation. Furthermore, the availability of free thiol groups in anti-human HMGB-1 IgG antibodies post-UV irradiation and subsequent borophene binding was assessed by the Ellman assay. A standard calibration curve generated using L-cysteine concentrations ranging from 0 to 200 nM yielded a linear correlation (*R* ^2^ = 0.9959) (Figure S9). Samples of anti-human HMGB-1 monoclonal antibodies (50 μg/mL) were diluted in reaction buffer for analysis. UV-untreated control antibodies exhibited minimal absorbance (*A*_412_ ∼ 0.009), similar to the Ellman’s reagent blank, confirming the absence of accessible thiol groups in the native antibody structure. Following photoinduced thiol generation via exposure to 254 nm UV irradiation (Trylight UV lamp, 6 W; irradiance ∼ 0.3 W/cm^2^ for 30 s), a significant increase in absorbance was detected (*A*_412_ ∼ 0.315), corresponding to 20.58 nM free thiol groups generated from disulfide bond cleavage. After incubation with borophene, absorbance decreased to 0.125, indicating 10.73 nM free thiol, a reduction of approximately 48%, suggesting substantial thiol–borophene binding, likely through B–S interactions. This thiol-specific interaction likely results in the antibodies adopting a side-on orientation on the borophene nanosheets, leaving only one Fab arm accessible for antigen binding (Figures S7 and 2a). Consequently, although IgG structurally possesses bivalent antigen-binding sites, the functional valency is effectively reduced to monovalent binding due to steric constraints imposed by the borophene nanosheet surface.

### Antibody-Functionalized Borophene Nanosheets for Biosensing Applications

It was essential to confirm the antibodies’ conjugation and orientation on the surface following the successful functionalization of the borophene nanosheets. To achieve this, we developed an immunoassay-based methodology utilizing a mouse anti-human HMGB-1 IgG capture antibody (capture Ab), HMGB-1 antigen (HMGB-1 Ag), a biotinylated mouse anti-human detection IgG antibody (detection Ab), and streptavidin-coated gold nanoparticles (Strp-AuNPs). In this approach, 100 μL of borophene nanosheets conjugated with anti-Human HMGB-1 IgG capture Ab were incubated with 100 μL of HMGB-1 Ag at ambient conditions in a microfuge tube for 5 min. Thereafter, 10 μL of Strp-AuNPs conjugated with detection Ab were introduced to the solution and incubated at ambient temperature for 15 min. Following each incubation phase, centrifugation was used to remove unattached or free-floating antigens or antibodies from the solution matrix. We hypothesized that the successful conjugation of borophene nanosheets with capture Ab would enable the formation of a sandwich complex in the presence of anti-Human HMGB-1 IgG Ag. However, if the borophene nanosheets were not conjugated with the captured IgG antibodies, the sandwich complex would not form. We also used a control sample where the borophene sheets were mixed with UV-untreated anti-Human HMGB-1 IgG capture Ab. In this case, we hypothesize that the antibodies will not conjugate onto the surface of nanosheets, and a sandwich will not be formed. To validate our hypothesis, we employed TEM, AFM, and Raman spectroscopy. The TEM image revealed a borophene nanosheet, identifiable as a lighter-colored, semitransparent structure (Figure 2c). Superimposed on the nanosheet are spherical, darker regions representing gold nanoparticles (AuNPs), as shown in Figure 2c. These nanoparticles are conjugated via a sandwich complex composed of capture Ab, HMGB-1 Ag, detection Ab, and Strp-AuNPs. The image provides visual confirmation of the successful conjugation of borophene nanosheets with the capture of IgG antibodies, as evidenced by the formation of the sandwich complex in the presence of the HMGB-1 antigen. The distinct contrast between the nanosheet and the spherical nanoparticles highlights the functionalized borophene surface’s structural integrity and the nanoparticles’ specific attachment through antigen–antibody-mediated interactions. Figure 2d shows an HR-TEM image of the borophene nanosheet, revealing distinct lattice fringes with a measured lattice spacing of 2.92 Å. Figure 2e shows a TEM image of the control sample, wherein the gold nanoparticles are not superimposed on the borophene nanosheet. This observation arises from the UV-untreated capture antibodies, which lack the thiol groups necessary for conjugation to the borophene surface. Consequently, the gold nanoparticles aggregate independently rather than adhering to the borophene nanosheets, thereby confirming the absence of a sandwich structure. Furthermore, low and high-magnification HAADF-STEM images of the same sample revealed the borophene nanosheet as a light-colored sheet-like structure. In contrast, the dark spherical structures were identified as gold nanoparticles (Figure 2f). This was further validated through EDX, which confirmed the presence of boron exclusively in sheet-like structures. On the contrary, gold was exclusively detected within the spherical formations (Figure 2g). We also utilized AFM to study the formation of the sandwiched complex on the surface of borophene conjugated with capture antibodies. The sample containing borophene nanosheets conjugated with capture Ab was mixed with HMGB-1 Ag and Strp-AuNPs labeled with detection Ab. The resulting mixture was drop-cast onto cleaved mica to analyze the structural modifications of the 2D nanosheets induced by the antibody molecules using AFM. From the AFM images, the change in height due to the formation of the sandwich complex on top of the antibody-conjugated borophene nanosheets was distinguishable in comparison with pristine borophene. The pristine borophene nanosheets exhibited a surface height of 2.0 ± 0.5 nm, as observed from the height profile (Figure 2h). Upon the formation of the sandwich complex on top of the antibody-conjugated borophene nanosheets, a height increase to 3.5 ± 0.7 nm was observed, indicating potential successful attachment of the antibody molecules. The surface analysis further revealed the accumulation of sandwich complexes on top of the antibody-conjugated borophene nanosheets. Furthermore, with XPS analysis, the species detected in high-resolution spectra on the borophene sample included: CH_*x*_ (carbon species), C–O, C–N, C=O, CF_2_, sulfonates, reduced sulfur, boron, oxidized boron, and fluorides (Table S1). The expected composition of tryptophan and cysteine is presented in Table S2 for comparison. The S 2p core-level spectrum provides critical insights into sulfur’s bonding environment and oxidation state. Typically, the S 2p signal appears as a doublet (S 2p_3/2_ and S 2p_1/2_) due to spin–orbit coupling, with a characteristic energy separation of approximately 1.18 eV. The precise binding energy varies depending on the chemical state of sulfur and generally falls within the range of ∼161–170 eV. Thiols are generally found at 163.5–164.0 eV in the S 2p spectrum, while metal sulfides/disulfides and metal–sulfur bonds are generally found to have lower binding energy (161–162.5 eV). The formation of a boron–sulfur bond is indicated by a prominent peak at 163.3 eV, as confirmed by the XPS data (Figure 2i). This binding energy aligns with literature values (Table S3) for similar heteroatom-sulfur covalent bonds.^57^ The clear distinction between this peak and those representing free thiols (R–SH at 164.6 eV) and oxidized sulfur species (SO_3_ at 167.86 and 169.04 eV) further supports our interpretation. The significant intensity of the B–S peak relative to other sulfur species indicates substantial covalent interaction rather than mere physical adsorption. Selected binding energies are presented in Table S3. These findings demonstrate that the capture antibodies were covalently attached to the borophene surface via thiol moieties generated through UV irradiation, highlighting the effectiveness of the functionalization strategy and its potential application in immunoassays.

### Analysis of Molecular Interaction and Antibody Orientation

To gain deeper insight into the molecular interactions occurring at the interface upon UV irradiation of antibodies, we leveraged the unique capabilities of surface-enhanced Raman spectroscopy (SERS). In this study, we employed a gold substrate as the plasmonic surface. IgG antibodies, UV-treated and untreated, were immobilized using a custom micropipette system designed to deliver a controlled flow of solution across the surface (see Materials and Methods for details). Following deposition, the samples were analyzed via a Raman spectrometer. The resulting SERS spectra (Figure 3a) were compared to the conventional untreated antibody. We observed significant alterations in the intensity of S–S stretching bands in the UV-treated IgG compared to the untreated control. A distinct spectral shift near 517 cm^–1^, corresponding to the Cys76–Cys94 disulfide bridge, suggests the emergence of a trans Ca–S conformation, potentially associated with an increase in free thiol generation.^58^ Additional shifts at 504, 514, 522, 527, and 544 cm^–1^ in the UV-irradiated samples indicate conformational changes likely related to the cleavage or rearrangement of disulfide bonds. These changes, attributed to the cystine residues, exhibit characteristic stretching vibrations within the 505–550 cm^–1^ spectral range. gauche–gauche-gauche (ggg) conformations have an S-S stretching band at ∼505–515 cm^–1^, gauchegauche–trans (ggt) have an S-S stretching band at ∼ 520–530 cm^–1^, trans-gauche–trans (tgt) have an S-S stretching band at ∼540–545 cm^–1^.^59^ These observations support the hypothesis that UV exposure promotes the photoreduction of disulfide bridges, forming free thiol groups that can interact more readily with the gold surface. Raman spectroscopy is particularly well-suited for distinguishing between reduced (free thiol) and oxidized (disulfide) states. The disappearance of S–S vibrational bands (e.g., at 503 cm^–1^) alongside the emergence of thiol-associated signals (e.g., around 680 cm^–1^) is^38^ characteristic of disulfide bond reduction. (Figure 3b). Aromatic amino acids are highly responsive to plasmonic enhancement and typically produce strong signals in protein Raman spectra. In the UV-treated sample, signals from phenylalanine (Phe), tyrosine (Tyr), and tryptophan (Trp) are significantly amplified. Notably, the characteristic Phe bands at 1122, 1240, 1335, and 1450 cm^–1^ show marked intensity increases, likely due to their closer proximity to the plasmonic surface (Figure 3c). Similarly, enhanced Tyr signals are observed at 830, 850, and 875 cm^–1^ (Figure S10). The overall SERS results indicate that the illumination parameters used for PIT effectively facilitated thiol-mediated surface attachment of the Ab molecules, more so than in the untreated Ab samples. Additionally, the enhanced intensity of numerous vibrational modes in the UV-treated samples suggests that antibody anchoring may also involve broader contact with the surface.

**Figure 3:**
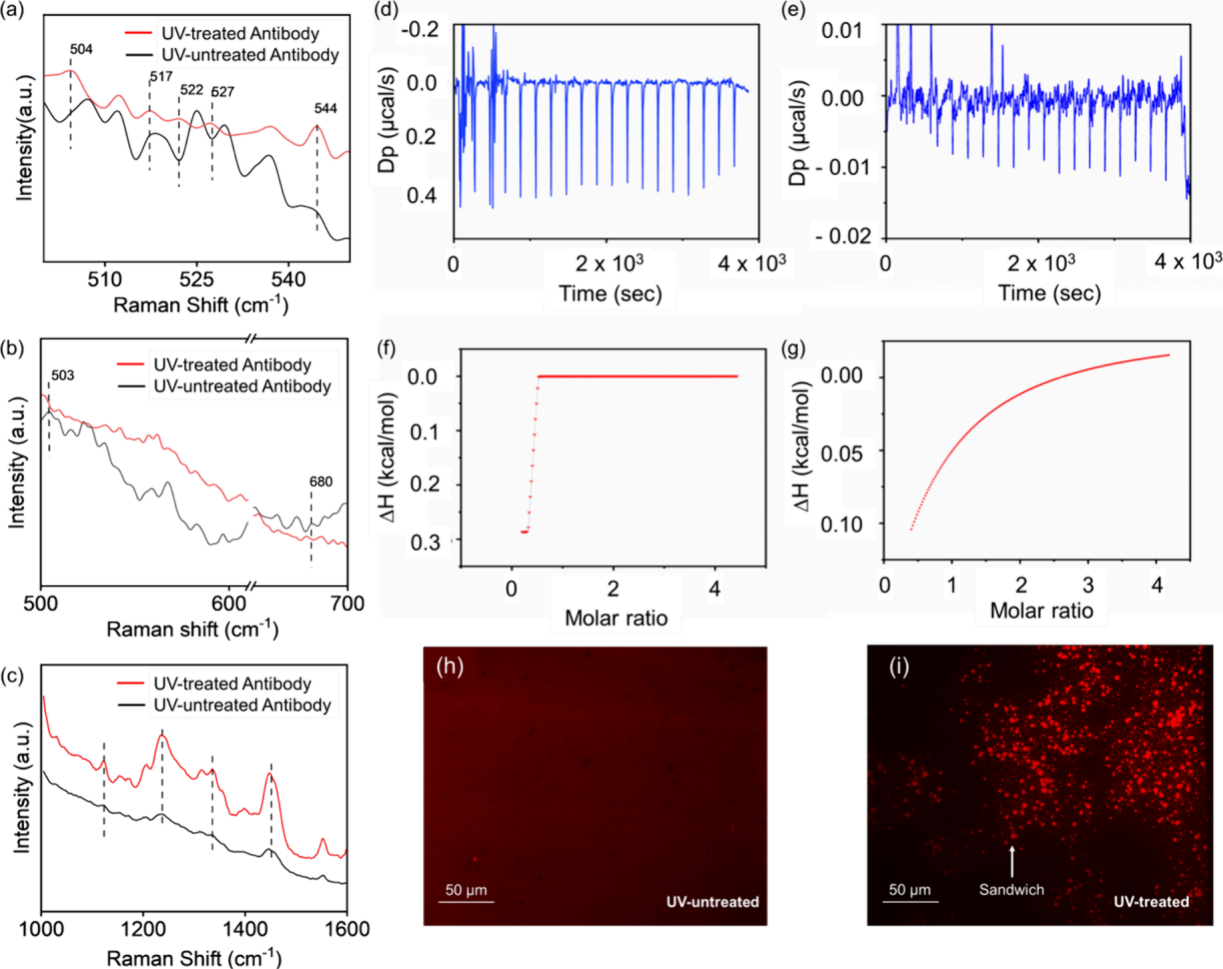
(a–c) Raman spectral analysis: (a) Signal intensity changes in the S–S region of 500–550 for the UV-treated antibody, indicating the reduction of the disulfide bond to produce the free thiol groups. (b) Disappearance of S–S vibrational bands (e.g., at 503 cm^–1^) alongside the emergence of thiol-associated signals (e.g., around 680 cm^–1^),^38^ which is characteristic of disulfide bond reduction. (c) Raman spectral analysis reveals distinct changes in the phenylalanine for the UV-treated antibody. (d–i) Isothermal titration calorimetry (ITC) and fluorescence analysis of borophene nanosheets with UV-treated and UV-untreated antibodies: (d) ITC thermogram of borophene nanosheets with UV-treated antibodies. (e) ITC thermogram of borophene nanosheets with UV-untreated antibodies. (f) Binding isotherm of borophene nanosheets with UV-treated antibodies. (g) Binding isotherm of borophene nanosheets with UV-untreated antibodies. (h) No fluorescence was observed for borophene nanosheets with UV-untreated antibodies, indicating a lack of antibody binding and subsequent Cy5 attachment. (i) Representative fluorescence image at 100 nM PfLDH concentration showing red spots corresponding to fluorescence emitted by single Cy5 molecules.

Moreover, we employed isothermal titration calorimetry (ITC) to investigate the thermodynamic interactions of anti-Human HMGB-1 IgG capture antibodies immobilized via PIT on 2D borophene nanosheets, compared to UV-untreated anti-Human HMGB-1 IgG antibodies on the same nanomaterial. Briefly, a 50 μM solution of borophene nanosheets was loaded into a syringe for subsequent addition to the reaction cell containing either the UV-treated or untreated anti-Human HMGB-1 IgG capture antibodies. Further, the concentration of HMGB-1 antibodies (both UV-treated and untreated) in the reaction cell was maintained at 1 mM for all titrations. The ITC thermograms and binding isotherms as shown in Figure 3 demonstrate a distinct difference in the binding characteristics of UV-treated and UV-untreated IgG antibodies on the borophene nanosheet surface. For UV-treated IgG (Figure 3d, 3f), the thermogram exhibits sharp, well-defined peaks, indicative of a strong and favorable interaction. The binding stoichiometry (*n*) is 0.318, suggesting partial occupancy of available binding sites, with a dissociation constant (*K*_D_) of 1.00 × 10^–9^ M, reflecting high binding affinity. The enthalpy change (Δ*H*) is 0.287 kcal/mol, consistent with minimal heat release, while the Gibbs free energy (Δ*G*) is highly favorable at −42.14 kcal/mol, confirming a thermodynamically strong binding interaction.

In contrast, for UV-untreated IgG (Figure 3e, 3g), the thermogram reveals smaller, less defined peaks, suggesting a weaker and less favorable binding process. The binding stoichiometry is significantly lower (*n* = 0.100), and *K*_D_ increases to 4.29 × 10^–5^ M, indicating a much weaker interaction. The Δ*H* value is higher at 3.871 kcal/mol, signifying a greater heat release during binding, but Δ*G* is reduced to −32.97 kcal/mol, confirming the relatively lower binding affinity. Collectively, these parameters indicate two distinct binding modes: the first reaction is rapid, enthalpically driven, and exhibits highly favorable free energy, while the second interaction is slower, presumably influenced by the entropic factors, and features significantly reduced affinity. The stoichiometric values suggest that both systems may involve cooperative or heterogeneous binding events. Thus, the results reveal that UV treatment significantly enhances the binding affinity of IgG antibodies to borophene nanosheets, likely through the formation of boron–thiol covalent bonds.

After confirming the effective adhesion of UV-treated IgG antibodies to the borophene nanosheet surface, the subsequent objective was to determine their orientation. Minopoli et al. previously employed fluorescence microscopy to validate the orientation of antibodies achieved through PIT.^56^ In their work, UV-induced cleavage of disulfide bonds in anti-*P*LDH antibodies—targeting the malaria antigen *Plasmodium* lactate dehydrogenase (*P*LDH)—produced thiol groups that covalently anchored the antibodies to a gold substrate. This approach ensured that at least one of the two Fab regions remained exposed. The accessibility and functionality of these Fab regions were verified via fluorescence measurements after the binding of a labeled target antigen (a*P*LDH-Cy5 aptamer). To confirm antibody orientation on borophene nanosheets, we adopted a similar strategy using anti-*P*LDH IgG antibodies, *P*LDH antigen, and a Cy5-labeled aptamer specific to *P*LDH. The PIT technique was employed to introduce thiol groups into IgG antibodies, which were then conjugated to the borophene nanosheets. The resulting antibody-functionalized nanosheets were incubated with *P*LDH antigen bound to a Cy5-labeled aptamer. The experimental design included borophene nanosheets functionalized with anti-*P*LDH IgG antibodies that serve as a substrate for capturing the malaria biomarker *P*LDH. The Cy5-labeled aptamer (5′-Cy5-CTG GGC GGT AGA ACC ATA GTG ACC CAG CCG TCT AC-3′) constituted the top layer, providing both fluorescence labeling and high specificity for *P*LDH at a relatively low cost. Fluorescence microscopy was employed to visualize the fluorescence signal, and the resulting images were processed with Image J software to quantify the corresponding signal intensity. As shown in Figure 3h, borophene nanosheets conjugated with UV-untreated antibodies exhibited no discernible fluorescence signal in the background-corrected image, indicating limited antigen-binding capacity. In contrast, borophene nanosheets functionalized with UV-treated antibodies display distinct red fluorescence spots (Figure 3i), corresponding to photons emitted by individual fluorophores. These results confirm that the PIT strategy facilitates the oriented immobilization of antibodies on borophene nanosheets and preserves the accessibility of their Fab regions for antigen binding.

### Assessment of Borophene Nanosheet–Antibody Conjugate Immobilization on Nitrocellulose Membrane and Antigen Capture

Building upon the previously obtained results, PIT was employed to functionalize 2D borophene nanosheets with anti-Human HMGB-1 IgG antibodies, and the feasibility of converting the immunoassay (detailed in Materials and Methods) into an LFIA format was examined. Before utilizing the borophene nanosheet-antibody conjugates, we wanted to conduct specific stability tests to ensure that borophene remains stably bound during assay conditions. Using a micropipette, borophene was drop-cast onto the nitrocellulose (NC) strip at a 0.175 mg/mL concentration. The strips were assembled by laminating the NC–borophene layer with a sample pad and an absorbent pad on a backing card. Critically, a standard assay using chase buffer was performed to assess whether physically entrapped borophene would be washed away during lateral flow. As evidenced by photographs taken before and after the assay (Figure S11), the borophene remained visibly intact in its original position on the membrane following fluid flow, confirming the stability of this physical immobilization approach. With the immobilization stability confirmed, we next evaluated the efficiency of borophene nanosheets–antibody conjugate deposition and its impact on antigen capture performance. To this end, both UV-treated and UV-untreated anti-Human HMGB-1 IgG capture antibodies were mixed with borophene nanosheets and deposited onto nitrocellulose membranes of defined dimensions. Specifically, the nitrocellulose membrane was sectioned into strips of 3 mm size, and 0.5 μL of borophene–antibody conjugates (UV-treated and conventional) were applied to each membrane at a concentration of 0, 25, 50, and 75 μg/mL. The borophene–antibody conjugates were designated as the test zone (T). After the spotting procedure, the membranes were allowed to dry at 37 °C. Once dry, the membranes were assembled into lateral flow test strips and analyzed for HMGB-1 antigen detection following the procedures detailed in Materials and Methods. As shown in Figure S12, test strips containing the UV-treated antibody–borophene nanosheet conjugates exhibited stronger signals than conventional LFIA. This enhancement can be attributed to improved antibody orientation and increased availability of Fab fragments for antigen binding. As previously discussed, one of the intrinsic limitations of lateral flow assays arises from the random orientation of adsorbed antibody molecules, which reduces their antigen-binding capacity and thus diminishes assay sensitivity. By utilizing the PIT strategy in conjunction with borophene nanosheets, it was possible to address this orientation issue and enhance the overall sensitivity of the lateral flow immunoassay.

### Characterization of the Immobilized Borophene–Antibody Conjugates on Nitrocellulose Membranes

Having established the feasibility of integrating the HMGB-1 immunoassay into a lateral flow assay format, the next step involved the development of a fully operational lateral flow immunoassay. To ensure optimal performance, it was necessary to investigate and characterize the molecular interactions occurring on the nitrocellulose membrane surface, particularly those involving the immobilized borophene–antibody conjugates. This was accomplished by designing a sandwich immunoassay in a lateral flow format incorporating gold nanoparticles. In brief, anti-Human HMGB-1 IgG antibodies (50 μg/mL) subjected to UV treatment were mixed with borophene nanosheets at (0.175 mg/mL) and incubated at room temperature for 5 min. As a control, borophene nanosheets were combined with UV-untreated anti-Human HMGB-1 IgG antibodies, coated onto a nitrocellulose membrane using an antibody striping machine, and then dried in an incubator at 37 °C for 2 h. The lateral flow assay was subsequently assembled following the protocols detailed in Materials and Methods. As shown in Figure S13, the assay incorporating UV-treated antibodies generated a significantly stronger signal than strips lacking UV-irradiated antibodies, indicating successful covalent linkage of UV-treated antibodies to borophene nanosheets. These results suggest that UV-untreated antibodies do not effectively adhere to the borophene surface during the assay, likely due to the absence of thiol groups necessary for stable binding.^32^ We utilized scanning electron microscopy (SEM) to investigate the role of 2D borophene nanosheets in increasing the surface area of nitrocellulose membranes and facilitating the formation of a sandwich structure in the lateral flow assay’s test zone. From our previous work, depositing an aqueous borophene suspension onto a glass slide and allowing it to evaporate yielded a heterogeneous array of rough-edged microparticles of varying sizes (Figure S14). At higher magnification, the SEM images revealed that these larger borophene sheets consisted of smaller aggregates, which in turn were formed by the aggregation and stacking of nanometer-scale particles, indicating a hierarchical structure. Building on these findings, we coated the test line zone of the nitrocellulose membrane with 2D borophene nanosheets and compared it with a plain NC membrane using SEM analysis (Figure 4a–d). The plain NC membrane exhibited a three-dimensional open-pore configuration composed of interconnected fibrous threads, along with spherical features measuring 2–4 μm in diameter fused onto these fibrous structures (Figure 4a). In contrast, the region coated with 2D borophene nanosheets displayed evidence of borophene sheets (Figure 4b), suggesting that these nanosheets predominantly accumulate on the top layer of the nitrocellulose pore network. Furthermore, cross-sectional SEM images of both borophene-coated and plain NC membranes were acquired at various magnifications (Figure S15). Although individual nanosheets could not be discerned, the data indicate that borophene particles primarily permeate the top 60–70 μm of the 130 μm-thick NC membrane (Figure S15). Collectively, these SEM observations provide qualitative evidence that the borophene nanosheets enhance the surface area of the nitrocellulose strips’ top layer. Elemental analysis via EDX conducted on different regions of the NC membrane showed an average composition of 54.35% carbon (Figure 4c), 29.72% boron (Figure 4d), 6.90% nitrogen, and 4.80% oxygen (Table S4 and Figure S16 ).

**Figure 4:**
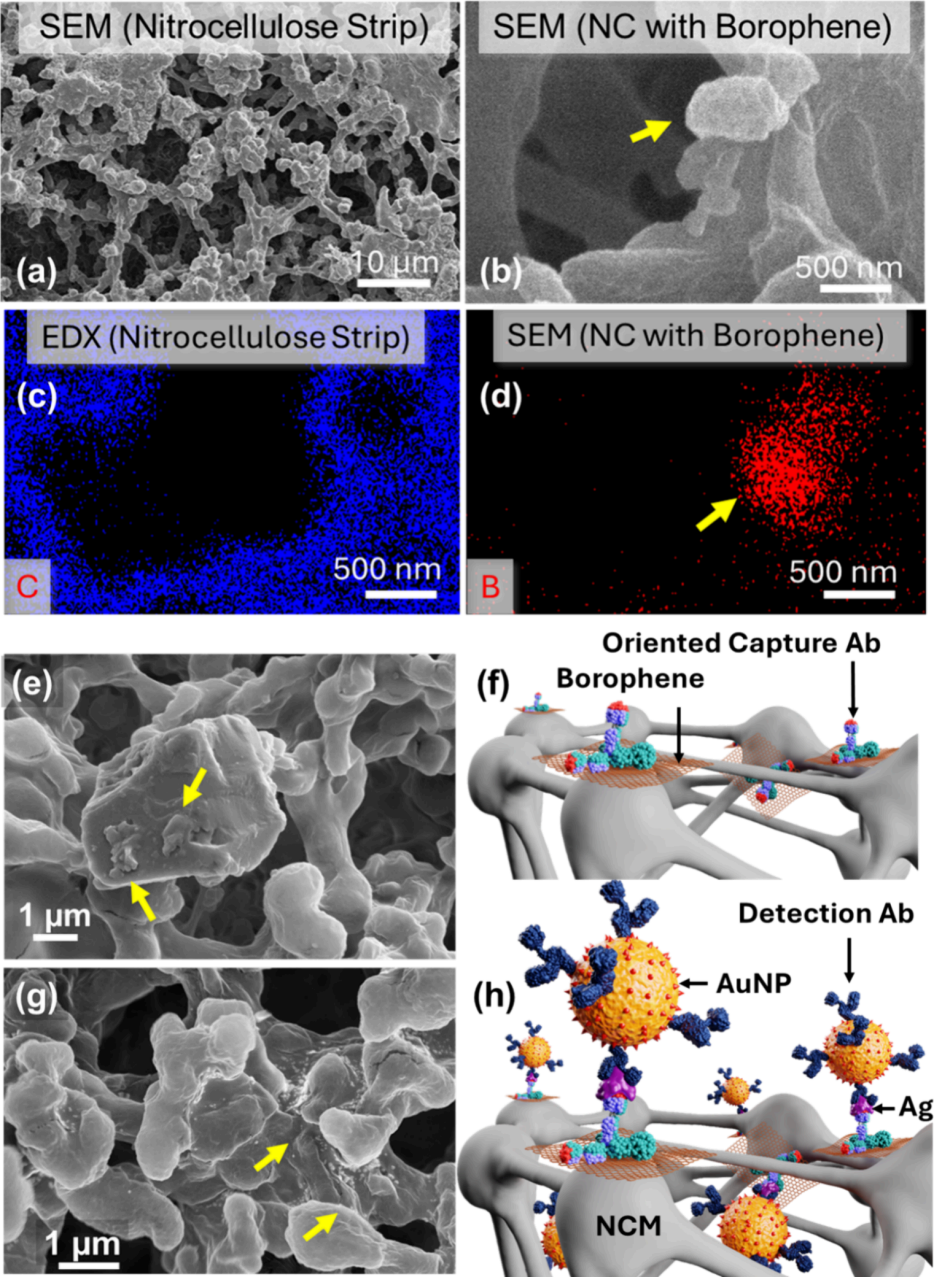
(a, b) SEM images comparing (a) a bare nitrocellulose (NC) membrane and (b) an NC membrane incorporated with borophene nanosheets. (c, d) Elemental mappings showing (c) the presence of carbon in the NC membrane and (d) the boron distribution in areas where borophene nanosheets are on the NC membrane. (e) SEM image of an NC membrane striped with a borophene–antibody conjugate. Arrows indicate the region containing the conjugates. (f) Schematic representation of the borophene–UV-treated antibody conjugate within the NC matrix, illustrating the oriented antibodies on the borophene nanosheet surface. (g) SEM image of the NC membrane after the formation of the sandwich complex with the NC matrix. Arrows indicate the presence of the sandwich complex as identified by the presence of gold nanoparticles. (h) Schematic illustration showing the formation of the sandwich complex within the NC matrix.

As discussed previously, the sensitivity of LFIAs is constrained by the orientation of the capture antibodies within the nitrocellulose membrane. Beyond orientation challenges, the capture molecules in the NC matrix tend to distribute uniformly throughout the membrane’s thickness (approximately 120–180 μm) without forming any noticeable gradient. Consequently, analyte recognition occurs at the membrane surface and within the three-dimensional open-pore structure (∼3–20 μm) of the NC matrix. Therefore, a substantial fraction of the resulting detection signal may be concealed from automated readers or operators due to the membrane’s opacity, leading to diminished signal intensities and higher detection limits compared with scenarios where all binding events occurred at the surface. To address this limitation, the introduction of a 2D nanomaterial–antibody conjugate with a high surface-to-volume ratio as the detection element in the NC strip’s test region could potentially enhance both the immobilization density and the antigen–antibody interaction efficiency nearer to the membrane surface, thereby improving the assay’s overall sensitivity.

Further, the SEM images showed that the immobilizing UV-treated anti-Human HMGB-1 IgG antibodies over the 2D borophene nanosheets on the test line region of the NC membrane showed a densely packed arrangement of antibodies (Figure 4e). Figure 4f shows the graphical representation of the borophene–antibody conjugate in an NC matrix highlighting that the photoinduced immobilization strategy enables strong covalent anchoring of the antibody to the metal surface, positioning one Fab region side-on orientation while exposing the other Fab to the environment for effective antigen binding. Further, upon the addition of the HMGB-1 antigen, the sandwich is formed (Figure 4g,h). The sandwich structure consists of a borophene-capture antibody in the bottom layer followed by HMGB-1 antigen and then streptavidin gold nanoparticles labeled with biotinylated detection antibody (Figure 4h). The SEM image confirmed the presence of sandwich structure in the test zone (Figure 4g). Moreover, the sandwiched structures appeared to be formed in a concentrated area indicating the densely packed antibodies over the borophene surface (Figure 4g).

Moreover, we also utilized XPS to understand the chemical composition of the sandwich structure formed on the test line. Our group previously reported the conjugation of cysteine to borophene^32^ for chiral induction to the 2D material. The interaction of B atoms with the thiol group of cysteine results in B–S bond formation, which was established with XPS as provided (Figure S17). This knowledge was leveraged for the conjugation of antibodies to the borophene surface for lateral flow sensing technology. XPS analysis was carried out with the lateral flow nitrocellulose strip containing the borophene antibody sandwich and compared with the control strip without the sandwiched complex. The XPS spectra of the pristine nitrocellulose membrane (Figure S18) and borophene–antibody sandwich containing nitrocellulose membrane gave an understanding of the B, C, N, O, and S presence. The B 1s spectrum, while not visible in the control nitrocellulose surface (Figure S18), is present in the borophene antibody sandwich spotted nitrocellulose strip. Three prominent peaks were observed at 187.5 eV for B–B bonds, whereas the B–O bond at 200.5 eV^32,60^ (Figure 5a). The O 1s peaks for N–O are observed at 532.9 eV, C–O–N is observed in 534.2 eV with a higher intensity and C–O–C at 532.9 eV. A peak at 531.5 eV is observed for B–O from the borophene-attached moiety (Figure 5b). The high-resolution XPS data of the NC membrane shows the C 1s region with peaks at 284.6, 287.0, and 288.6 eV (Figure 5c). These peaks can be assigned to the C–C, C–O–C, and C–O–N bonds.^61^ The peak at 289.6 eV arises from the carboxyl group in the antibodies. The S 2p spectrum shows two peaks at 168.2 and 169.5 eV. The peak of N 1s for nitrocellulose is consistent with the NO_2_ peak at 407.9 eV (Figure 5d). For the borophene antibody sandwich on NC, two new peaks arise at 404.8 eV for NH_2_ and another peak at 400.3 eV for amide groups from the antibody.^62^ Raman spectra conformed the peaks appearing in nitrocellulose strip containing antibodies immobilized onto borophene nanosheets (Figure 5e). AFM analysis of the borophene-coated nitrocellulose (NC) membrane with the sandwich complex revealed an average roughness height (*S*_a_) of 1.23 nm and a root-mean-square (RMS) roughness (*S*_q_) of 1.72 nm (Figure 5f). In comparison, the NC membrane without the sandwich complex exhibited *S*_a_ and *S*_q_ values of 0.94 and 1.19 nm, respectively (Figure 5g). These changes in surface roughness are in agreement with the presence of immobilized biomolecules. However, we acknowledge that AFM alone cannot definitively resolve antibody orientation, particularly on topographically irregular surfaces like borophene. Therefore, while the increased roughness may suggest surface functionalization, the effective sandwich formation in the presence of HMGB-1 antigen could be due to the accessibility and binding functionality of the antibody Fab regions.

**Figure 5:**
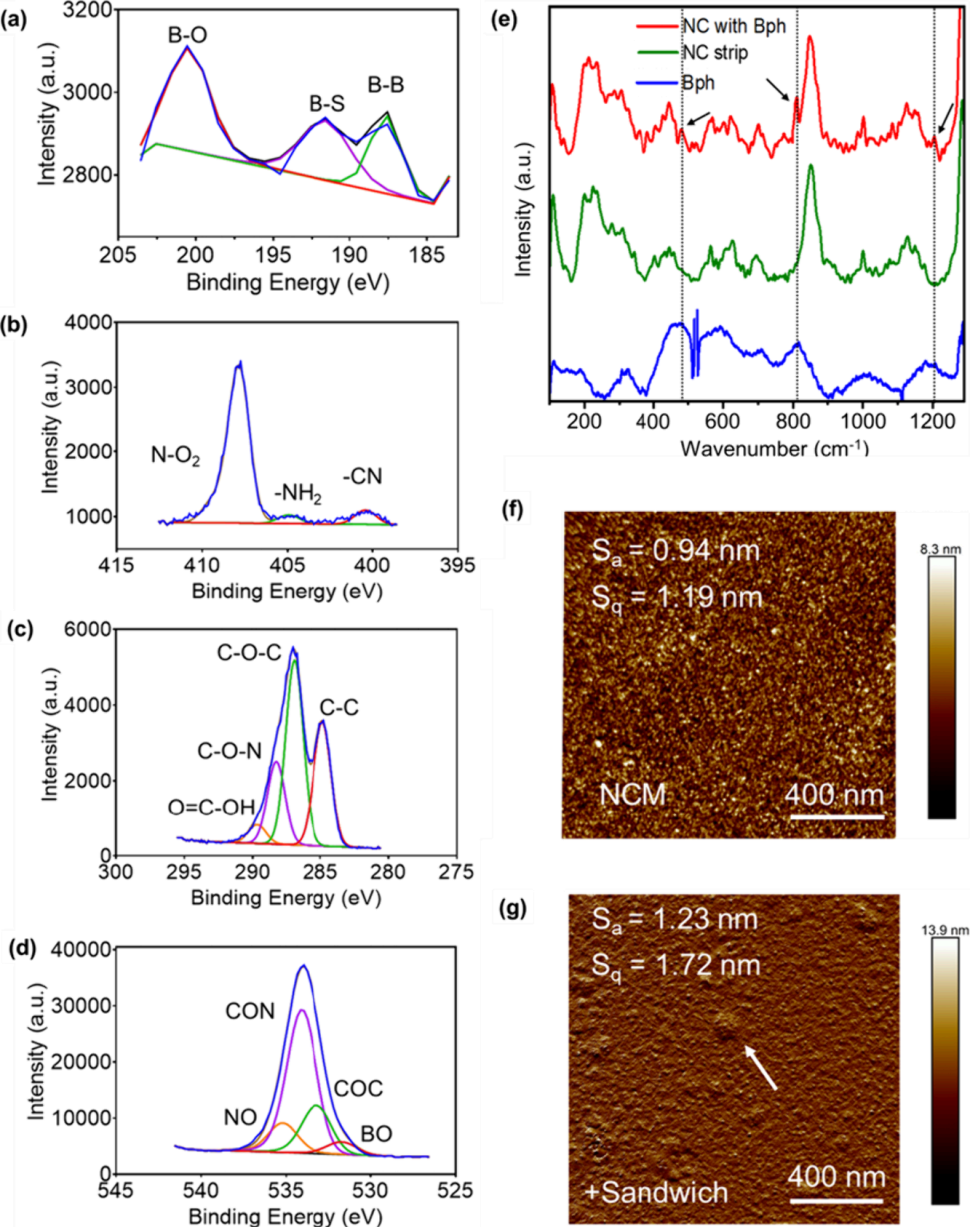
Deconvoluted XPS spectra showing the (a) boron peaks in B 1s (b) N 1s (c) C 1s, and (d) O 1s from the borophene content in the nitrocellulose (NC) membrane. (e) Raman spectra of the NC membrane and the NC membrane coated with borophene nanosheets. (f, g) Morphological analysis of the NC membrane surfaces used in LFIA, as characterized by AFM: (f) NC membrane; (g) test line on the NC membrane showing the sandwich complex of antigen, detector antibody, and capture antibody.

### Borophene–Antibody Conjugate Lateral Flow Assay for HMGB-1 Detection in Menstrual Effluent

Having characterized the molecular interactions at the test line of our developed lateral flow immunoassay, we proceeded to evaluate its capacity for detecting HMGB-1 in whole menstrual effluent. In this study, we employed photoinduced immobilization to covalently attach anti-Human HMGB-1 IgG capture antibodies onto 2D borophene nanosheets and compared the assay performance to that of a conventional LFIA in a sandwich-based format (Figure 6). In the borophene-based LFIA, the test line of the nitrocellulose membrane was coated with UV-treated anti-Human HMGB-1 IgG capture antibodies on 2D borophene nanosheets. In the conventional LFIA, only the HMGB-1 capture antibody was immobilized on the nitrocellulose membrane (Figure 6a). Both assays utilized the same Anti-mouse IgG at the control line and a conjugation pad containing gold-labeled anti-Human HMGB-1 IgG detection antibodies. Various concentrations of HMGB-1 (0–1000 pg/mL) were spiked into whole menstrual blood and applied to the sample pad, which incorporated a blood separator. The blood separator (a glass fiber filter pad) effectively separates blood components, trapping red blood cells and larger cellular debris on the surface while allowing only plasma containing the target HMGB-1 analyte and proteins to flow through to the nitrocellulose membrane (Figure 6b). This physical separation step significantly reduces matrix interference before the sample reaches the detection zone. As the sample flows, HMGB-1 binds to the gold-labeled detection antibodies, and this complex then migrates to the test line, where it interacts with the immobilized capture antibodies to form a visible sandwich complex. Unbound detection antibodies proceeded to the control line, yielding a separate signal. Visual analysis of the borophene-based LFIA indicated that test lines could be observed at HMGB-1 concentrations as low as 50 pg/mL (Figure 6d), whereas the conventional LFIA (Figure 6c) required at least 250 pg/mL to produce a visible band. These findings suggest that the borophene-based LFIA achieves a naked-eye detection limit that is approximately 80% lower (i.e., more sensitive) than the conventional assay. Subsequently, to evaluate potential cross-reactivity, the specificity of the lateral flow assay was examined using albumin, fibrinogen, and gamma globulin—proteins commonly present in whole menstrual effluent. Each protein (20 μg/mL) was individually mixed with 120 μL of chase buffer and applied to the lateral flow assay strip. None of these proteins elicited a signal at the test line as shown in Figure 6e. These observations confirmed the specificity of the lateral flow assay, which exclusively recognized its target recombinant protein HMGB-1 and did not interact with other recombinant proteins found in menstrual effluent. Furthermore, quantitative assessments were performed using an ESE Quant Flex reader, and the color intensities at the test line were analyzed with Studio 4.0 software. As expected, the signal intensities increased for both LFIA formats in proportion to increasing HMGB-1 concentrations. Figure 6f shows the calibration curves for the borophene-based and conventional LFIAs, which yielded high correlation coefficients (*R* ^2^ = 0.9772 and 0.9531, respectively). To calculate the analytical limit of detection of the borophene LFIA, we use LOD = 3.3*s*_*y*_/*y*. The LOD was approximately 40 pg/mL for the borophene-based LFIA and 240 pg/mL for the conventional LFIA. The rationale for targeting low concentrations, such as 40 pg/mL, is supported by clinical evidence indicating that HMGB-1 levels in menstrual blood are significantly elevated in individuals with endometriosis compared to healthy controls. However, early stage or asymptomatic cases may present with only modest increases. The incorporation of two-dimensional borophene nanosheets, combined with photoinduced antibody immobilization, enhances the assay’s analytical performance, resulting in an approximately 500% increase in sensitivity relative to conventional LFIA platforms. While ELISAs can detect lower HMGB-1 levels, our borophene-based LFIA achieves clinically relevant sensitivity (40 pg/mL) in a rapid point-of-care format, enabling noninvasive monitoring of localized inflammatory activity. This threshold aligns with reported menstrual fluid HMGB-1 levels in endometriosis progression, where early detection is critical for timely intervention.^63^ Unlike lab-based ELISA, our approach balances sensitivity with practicality for decentralized settings, addressing unmet needs in endometriosis screening.

**Figure 6:**
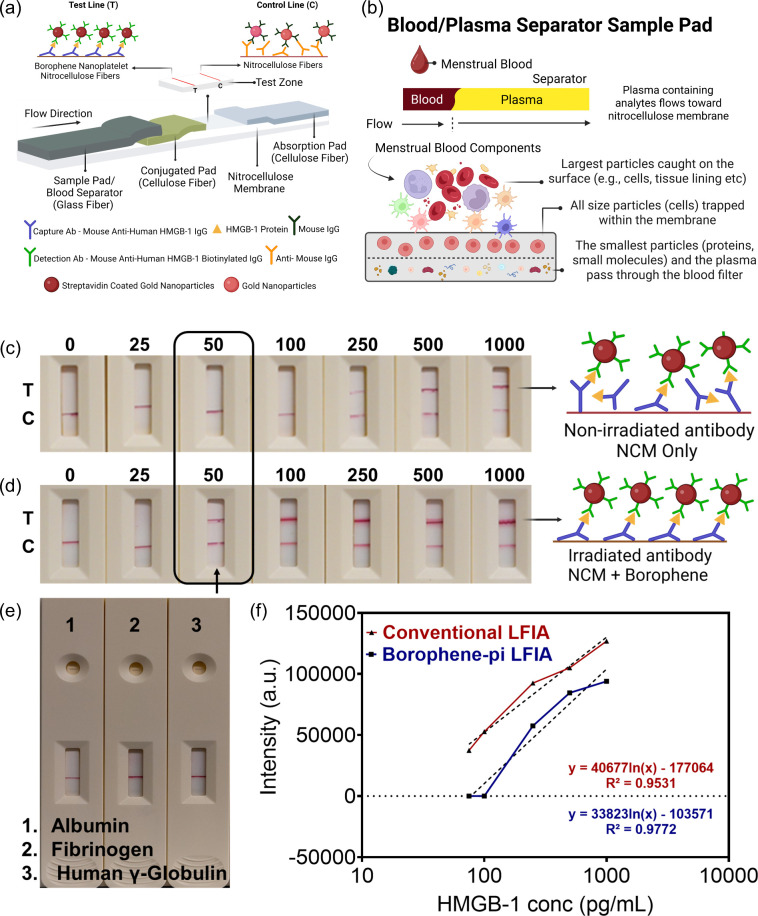
(a) Schematic representation of the developed borophene–antibody conjugate LFIA. (b) Schematic illustration representing the working principle for the blood separator. (c) Detection of HMGB-1 using a traditional LFIA with a limit of detection (LOD) of 250 pg/mL (d) Detection of HMGB-1 using the developed borophene–antibody conjugate LFIA with an LOD of 50 pg/mL, discernible to the naked eye. (e) Specificity of the developed borophene–antibody conjugate LFIA against commonly found proteins in menstrual effluent. (f) Calibration curves illustrating the comparative performance of the borophene–antibody conjugate LFIA against the conventional LFIA across a range of HMGB-1 concentrations from 0 to 1000 pg/mL under optimal experimental conditions.

## Conclusion

In this work, we have successfully developed a simple and efficient method for the immobilization of antibodies utilizing borophene combined with a unique functionalization procedure (PIT) to spatially orient antibodies on their surface to enhance their interaction with target analytes. This approach enabled the construction of a functional lateral flow assay to detect HMGB-1, a key biomarker for endometriosis in menstrual effluent at low concentrations. This success was achieved by exploiting the high surface-area-to-volume ratio of borophene and ensuring robust antibody orientation via photoactivation. The surface functionalization approach used in this platform yields spatially oriented antibodies, resulting in a densely packed distribution. This ensures optimal utilization of the biosensor’s interacting area, which may contribute to its exceptional sensitivity and an impressive detection limit of 40 pg/mL, surpassing conventional colorimetric assays. These findings tackle a key challenge in endometriosis diagnostics, where current methods often suffer from limited sensitivity. We expect that the proposed method will serve as a simple and universal platform for analyzing low-concentration analytes with high specificity and no cross-reactivity. Its clinical reliability makes it well-suited for real-time diagnostic and biosensing applications. A key innovation of this approach is the integration of LFIA strips into menstrual pads, enabling women to discreetly and conveniently monitor HMGB-1 levels at home. This advancement enhances accessibility to cutting-edge diagnostics, decentralizing the platform to empower patients while facilitating widespread use in resource-limited settings. Future research will focus on scaling up larger clinical studies, enhancing the device’s sensitivity, and expanding the assay to detect additional disease biomarkers. By integrating advanced 2D nanotechnology with user-friendly diagnostic formats, this borophene-based LFIA paves the way for more accessible, noninvasive, and highly precise detection strategies, with the potential to revolutionize reproductive health and beyond. Additionally, it highlights key research opportunities that must be explored to drive the development of next-generation biotechnologies centered on 2D materials.

## Materials and Methods

### Materials

All chemicals were purchased from Sigma-Aldrich (St. Louis, MO, USA) unless otherwise stated. HMGB-1 recombinant antigen, mouse capture anti-human HMGB-1 IgG, and biotinylated detection anti-human HMGB-1 IgG antibody (Z01LS-1122-LS68) were bought from Creative Biolabs Co., Ltd. (New York, NY, USA). Whole blood filter sample pad (Fusion-5, Cytiva, USA), an absorption pad (CFSP173000), and a high-flow nitrocellulose membrane (HF180) were bought from the Merck Millipore (Darmstadt, Germany).

### Synthesis of Borophene Nanosheets

A total of 250 mg of boron powder was dispersed in 250 mL of Milli-Q water in a beaker and thoroughly stirred. The mixture was then subjected to probe sonication for 10 h at a 12 μm amplitude, with a cycle of 2 s “on” followed by 1 s “off.” The resulting suspension was centrifuged at 3000 rpm for 3 min to collect the supernatant, which was then passed through a 0.45 μm PTFE filter and kept at 4 °C. The final concentration of the suspension was determined and subsequently used for further analysis.

### Synthesis of Gold Nanoparticles

Citrate-capped AuNPs were prepared by our previously published methods.^48–50^ Briefly, 8.5 mg of tetrachloroauric­(III) acid trihydrate (HAuCl_4_·3H_2_O) was dissolved in 95 mL of deionized water. This solution was transferred to a 200 mL round-bottom flask equipped with a reflux condenser, placed in an oil bath, and brought to a boil under magnetic stirring. Subsequently, 5.0 mL of a 1% (w/v) sodium citrate solution was added rapidly. The mixture was kept at a boil and stirred for 30 min until it developed a wine-red color. After cooling, the final product was stored in the dark at room temperature until further use.

### Streptavidin Conjugation to Gold Nanoparticles

To prepare a streptavidin conjugate with gold nanoparticles via electrostatic interaction, adjust the pH of 5 mL colloidal gold solution to 7.0 using a freshly prepared Na_2_CO_3_ solution. Add a streptavidin solution prepared in 10 mM potassium phosphate buffer (pH 7.2) to the adjusted colloidal gold solution. Incubate the mixture at room temperature while stirring for 30 min. Then, add BSA and sucrose to final concentrations of 10% and 0.2%, respectively, and incubate the mixture for 1 h at room temperature. Centrifuge the mixture at 11,000 rpm for 30 min at 4 °C using a 5810R centrifuge (Eppendorf, Germany). Discard the supernatant and dissolve the resulting pellet in 10 mM Tris-HCl buffer (pH 7.2).

### Dynamic Light Scattering

All samples were diluted to a concentration of 0.005 mg/mL in a disposable quartz cuvette. DLS measurements were carried out using a Malvern Instruments Zetasizer Nano series system equipped with a 633 nm laser. Each sample was measured three times, and the results were averaged using the Zetasizer software. For analysis, the refractive index of water was set to 1.33, and the viscosity was taken as 0.8872, with a PDI of approximately 0.1 for each measurement.

### Atomic Force Microscopy

AFM was carried out using a model 5600Ls atomic force microscope manufactured by Bruker Nano, USA. The analyses were performed in tapping mode in different sizes, using phase contrast and height modes. The raw images were processed with Nanoscope Analysis 3.0 imaging and analysis software package.

### HAADF-STEM Measurements

TEM images were taken using a Talos F200X microscope. HAADF-STEM images, EDX mapping, and EDX line-scan profiles were taken using an FEI 200 kV Titan Themis scanning transmission electron microscope.

### X-ray Photoelectron Spectroscopy

XPS experiments were performed using a Physical Electronics Versa Probe III instrument equipped with a monochromatic Al Kα X-ray source (*h*ν = 1486.6 eV) and a concentric hemispherical analyzer. Charge neutralization was performed using both low-energy electrons (<5 eV) and argon ions. The binding energy axis was calibrated using sputter-cleaned Cu (Cu 2p_3/2_ = 932.62 eV, Cu 3p_3/2_ = 75.1 eV) and Au (Au 4f_7/2_ = 83.96 eV) foils. Peaks were referenced to the CH_*x*_ band in the C 1s spectrum at 284.8 eV. Measurements were made at a takeoff angle of 45° concerning the sample surface plane. This resulted in a typical sampling depth of 3–6 nm (95% of the signal originated from this depth or shallower). Quantification was done using instrumental relative sensitivity factors (RSFs) that account for the X-ray cross-section and inelastic mean free path of the electrons. The analysis size was ∼100 μm in diameter.

### Raman Spectroscopy

#### Borophene Spectrum Acquisition

Raman spectra were collected using a Renishaw inVia Reflex Raman Spectroscope system with the following parameters: a 785 nm laser, 45 mW (50%) power, a grating of 1200, 100× magnification, and an acquisition time of 0.3 s, with the center of Raman frequency set at 1100 cm^–1^.

#### SERS Experiment

Raman measurements were performed using a Horiba LabRAM HR Evolution spectrometer equipped with a 633 nm excitation laser focused through a 100× objective lens (NA 0.9), delivering an incident laser power of 400 μW on the sample. Samples were deposited onto a gold (Au) substrate, and fast mapping was conducted with an integration time of 0.9 s, a confocal hole size of 100 μm, and a 300 gr/mm grating coupled to a BIDD Si-array detector (Horiba Synapse). The spectrometer was calibrated using the Raman response of a single-crystal silicon standard at 520 cm^–1^. Acquired spectra were processed by subtracting background signals using an eighth-order polynomial fit and subsequently averaged across all spectra obtained within the mapped region.

### FTIR Spectroscopy

FTIR in the attenuated total reflectance (ATR) mode was performed in an Agilent Cary 630 FTIR spectrometer from 4000 to 650 cm^–1^ at room temperature on a diamond detector.

### Scanning Electron Microscopy

The surface characterization of the test strip to ensure the establishment of conjugated probes on the NC membrane was performed using SEM. A Verios G4 scanning electron microscope was used to characterize the test strip NC membrane before and after loading of the HMGB-1 sample.

### Lateral Flow Test Strip Preparation

The lateral flow assay test strips were prepared by a Claremont antibody stripping machine and a Guillotine paper cutter. Photographs of strips and liquid samples in containers were taken using an ESI quant, and the Image was analyzed using Image Studio 4.0 The complete LFA strip consists of a blood filter sample pad, an absorbent pad, and a nitrocellulose membrane featuring one test (T) line and one control (C) line. These components were assembled on a backing pad with overlapping ends to ensure a continuous flow of the developing solutions. Biotinylated anti-Human HMGB-1 IgG antibodies conjugated with streptavidin-AuNP (1 μg/strip) were immobilized on the conjugation pad by incubation at 37 °C for 1 h. For the test and control lines, UV-treated anti-Human HMGB-1 IgG antibodies conjugated with borophene nanosheets and anti-mouse IgG antibodies were immobilized on the NC membrane, respectively. The anti-Human HMGB-1 IgG antibody-borophene conjugate was prepared by mixing 50 μg/mL UV-treated anti-Human HMGB-1 IgG with 0.175 mg/mL borophene nanosheets, followed by incubation at room temperature for 5 min. This conjugate was then sprayed onto the NC membrane using an antibody dispenser and dried at 37 °C for 1 h. The test and control lines were spaced 5 mm apart. The fully assembled LFA strip measured 3 × 60 mm and was stored in a sealed bag at room temperature until use. In conventional LFIA strip preparation, borophene is excluded from the test line, while the same nanoparticles are present on the conjugation pad.

### Detection of HMGB-1 Using Borophene-Based LFIA

A 10 μL sample of menstrual blood spiked with HMGB-1 standards was applied to the blood filter sample pad. After 30 s, 120 μL of PBST (1% BSA in PBS containing 0.05% Tween 20) was sequentially dispensed onto the pad. The sample then interacted with biotinylated anti-Human HMGB-1 IgG antibodies conjugated to streptavidin-AuNp complexes on the conjugation pad. Subsequently, the complex bound to borophene and the capture anti-Human HMGB-1 IgG antibody on the NC membrane, forming the test line, while unbound complexes migrated to form the control line. After approximately 15 min of incubation at room temperature, images of the strips were captured using an ESE Quant Flex. For quantitative analysis, the images were analyzed using Image Studio software, and the pixel intensity of the test line regions was measured to determine the color signal intensity.

### Bradford Assay

Borophene–antibody conjugation was performed by mixing 1 mL of borophene nanosheet suspension (0.175 mg/mL in ultrapure water) with anti-human HMGB-1 IgG antibody at a final concentration of 50 μg/mL. Prior to conjugation, the antibody solution was irradiated at 254 nm using a Trylight UV lamp (6 W, irradiance ∼ 0.3 W/cm^2^) for 30 s to generate reactive thiol groups. Following UV irradiation, antibodies were incubated with borophene for 3 min at 25 °C. The conjugation mixture was subsequently centrifuged at 3000*g* for 10 min at 4 °C to pellet the antibody-borophene complexes. The pellet was resuspended in 1 mL of ultrapure water for further analysis. A control group using nonirradiated antibodies was processed identically. To quantify antibody conjugation, a Bradford assay was performed using Coomassie Brilliant Blue G-250 dye. Briefly, IgG standards (1–100 μg/mL in phosphate-buffered saline (PBS), pH 7.4) and sample supernatants (150 μL) were mixed with 50 μL of Bradford dye reagent in a 96-well plate and incubated for 5 min at room temperature. Absorbance was measured at 595 nm using a BioTek Synergy H1 microplate reader. The linear standard calibration curve determined free IgG concentration in the supernatants. The conjugation efficiency (CE, %) was calculated using the following formula: CE = [1 – (Free IgG in supernatant/Total IgG initially added)] × 100%. The resulting conjugation efficiency was approximately 48%, corresponding to 24.04 μg/mL IgG immobilized on the borophene surface.

### Ellman Assay

The Ellman assay was used to quantify free thiol groups. The reaction buffer consisted of 0.1 M sodium phosphate buffer (pH 8.0) containing 1 mM EDTA. Ellman’s reagent (DTNB) was freshly prepared by dissolving 4 mg of DTNB in 1 mL of reaction buffer. A calibration curve was established using l-cysteine standards ranging from 0 to 200 nM. Anti-human HMGB-1 IgG antibodies were diluted to 50 μg/mL in the reaction buffer and exposed to UV irradiation (254 nm, Trylight UV lamp, 6 W, irradiance ∼ 0.3 W/cm^2^) for 30 s. A nonirradiated control group was maintained under identical conditions without UV exposure. Postirradiation, aliquots of 150 μL from each sample or standard were transferred into a 96-well plate, and 50 μL of Ellman’s reagent was added. The plate was incubated at room temperature for 15 min. After UV activation, antibodies were incubated with borophene nanosheets as described above. Postconjugation samples underwent the same Ellman assay procedure to assess residual free thiol concentration. Background absorbance from borophene alone was subtracted from all sample measurements. Absorbance values were recorded at 412 nm using a UV–vis spectrophotometer, and the concentration of free thiols was determined using the established standard curve.

## Supplementary Material



## Data Availability

The data supporting this study’s findings are available from the corresponding author upon reasonable request.

## Notes

DLS measurements for borophene nanosheets (Figure S1), SEM images for bulk boron and borophene nanosheets (Figure S2), TEM images and EDX mapping for borophene nanosheets (Figures S3 and S4), XPS spectra for bulk boron and borophene nanosheets (Figure S5), AFM image of borophene nanosheets (Figure S6), PIT illustration for antibody orientation (Figure S7), Bradford and Ellman assays (Figures S8 and S9), Raman spectral analysis for UV-treated HMGB-1 IgG antibodies (Figure S10), standardization of LFIA with borophene and UV-treated antibodies (Figures S11–S13), SEM analysis for borophene nanosheets immobilized in nitrocellulose membrane and corresponding elemental mapping (Figures S14–S16), XPS analysis of borophene nanosheets in nitrocellulose membrane (Figures S17 and S18), relative abundances of elements identified from XPS analysis on borophene nanosheets and UV-treated antibodies (Tables S1 and S2), B–S covalent bond appearance in XPS as per the literature (Table S3), and EDX data for the immobilization of the borophene over the NC membrane (Table S4) (PDF)
