# Supplementary material for: Photoinduced Immobilization on Two-Dimensional Nano Borophene Spatially Orients Capture Antibody for Highly Sensitive Biological Interactions
Source: ACS Cent Sci. 2025 Jul 17;11(8):1492–511. doi: 10.1021/acscentsci.5c00474 (PMC12395291; doi:10.1021/acscentsci.5c00474)
Supplement: Supplementary file 1 [file oc5c00474_si_001.pdf]

SUPPORTING INFORMATION

Photoinduced Immobilization on Two-Dimensional Nano Borophene Spatially Orients Capture Antibody for Highly Sensitive Biological Interactions

Satheesh Natarajan [a,1], Ketan Dighe [b,1], Teresa Aditya [a], Pranay Saha [a], David Skrodzki [c], Purva Gupta [b], Nivetha Gunaseelan [b], Shraddha Krishnakumar [b], and Dipanjan Pan [a,b,c,d,*]

[a] Department of Nuclear Engineering, The Pennsylvania State University, University Park, PA 16802, USA

[b] Department of Biomedical Engineering, The Pennsylvania State University, University Park, PA 16802, USA

[c] Department of Materials Science and Engineering, The Pennsylvania State University, University Park, PA 16802, USA

[d] Huck Institutes of the Life Sciences, University Park, PA 16802, USA

*Email of the corresponding author: dipanjan@psu.edu

**Table of Contents**	**Page number**
Figure S1	S3
Figure S2	S4
Figure S3	S5
Figure S4	S6
Figure S5	S6
Figure S6	S7
Figure S7	S7
Figure S8	S8
Figure S9	S8
Figure S10	S9
Figure S11	S10
Figure S12	S11
Figure S13	S12
Figure S14	S13
Figure S15	S14
Figure S16	S15
Figure S17	S16
Figure S18	S17
Table S1	S18
Table S2	S18
Table S3	S18
Table S4	S19

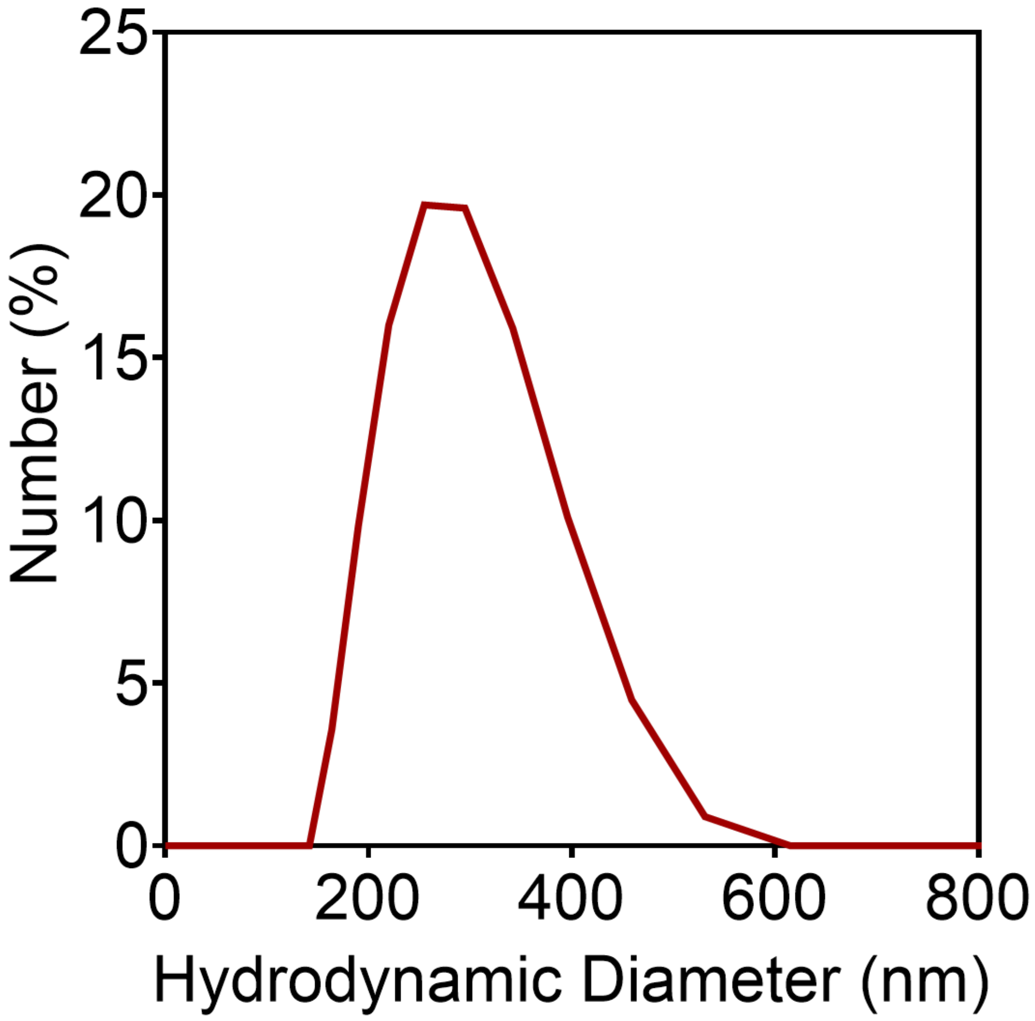

Figure S1. Dynamic Light Scattering (DLS) measurements of borophene nanosheets.

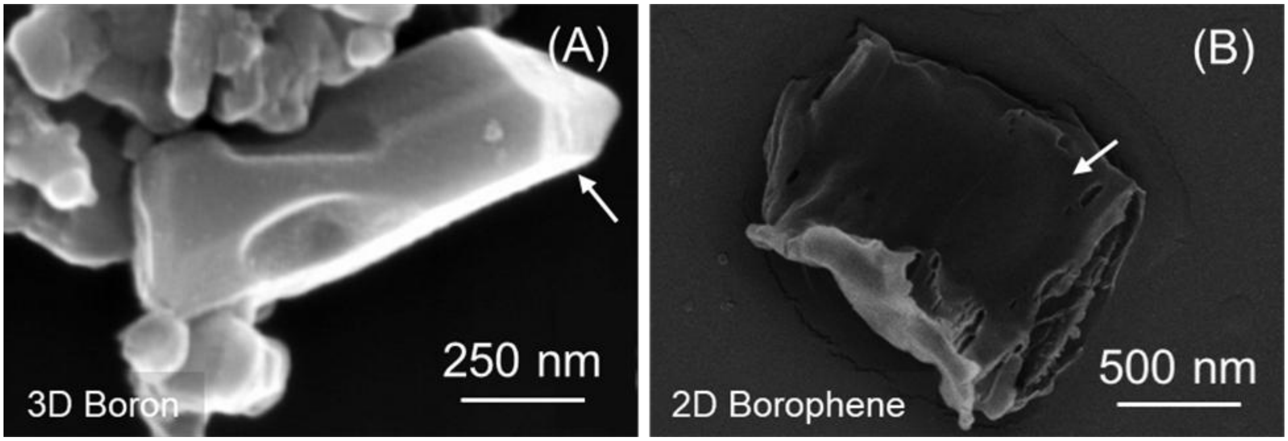

Figure S2. Scanning electron microscopy (SEM) images for **(A)** pristine boron powder precursor (Sigma-Aldrich) and **(B)** borophene nanosheets. The SEM images provide a comparison between the morphology of pristine boron powder (3D) and borophene nanosheets (2D) obtained after liquid exfoliation. Arrows indicate the 3D structure of boron and the 2D structure of the nanosheets.

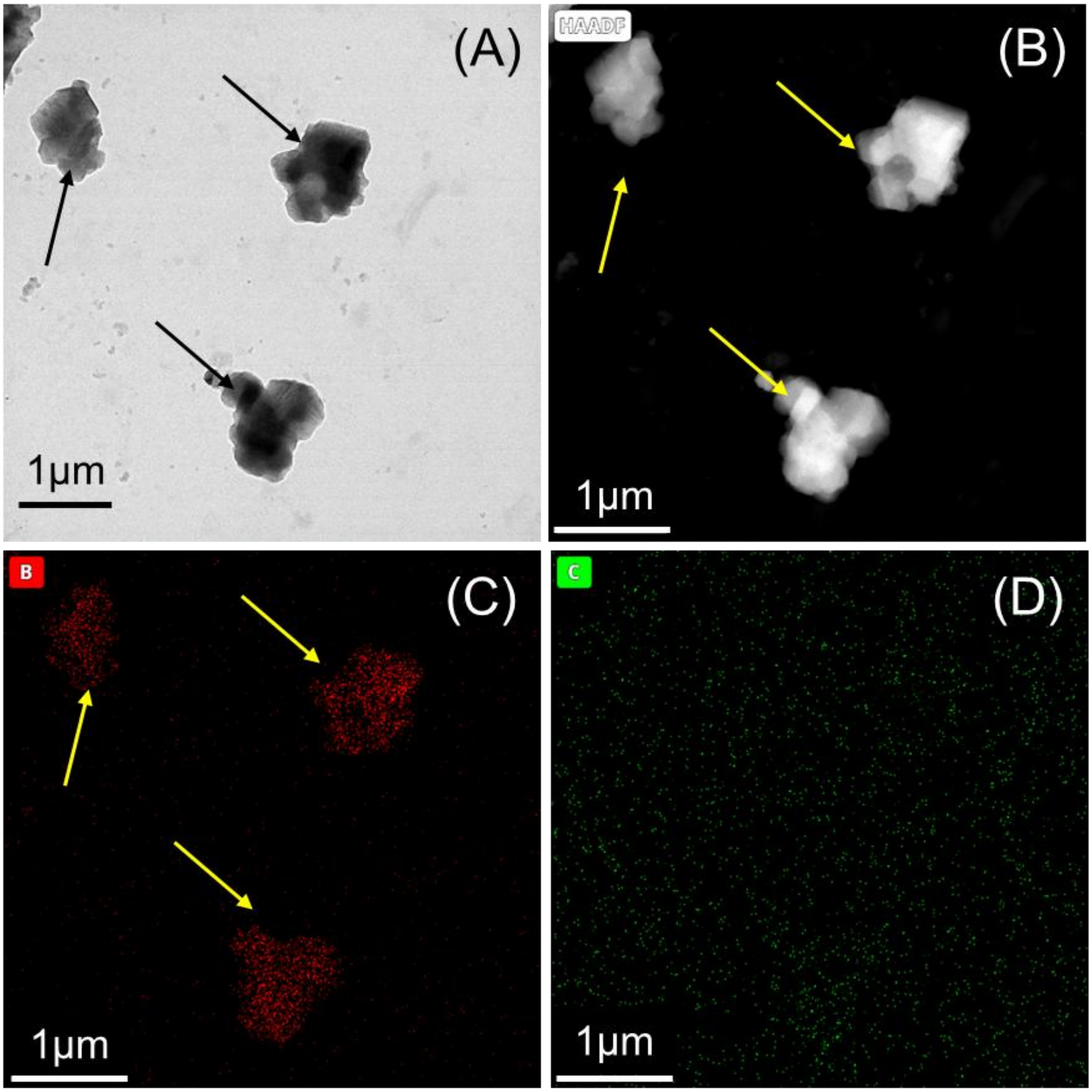

Figure S3. **(a)** TEM image (**b**) High-Angle Annular Dark Field (HAADF) image with elemental mapping (EDX mapping) (**c**) boron (**d**) carbon

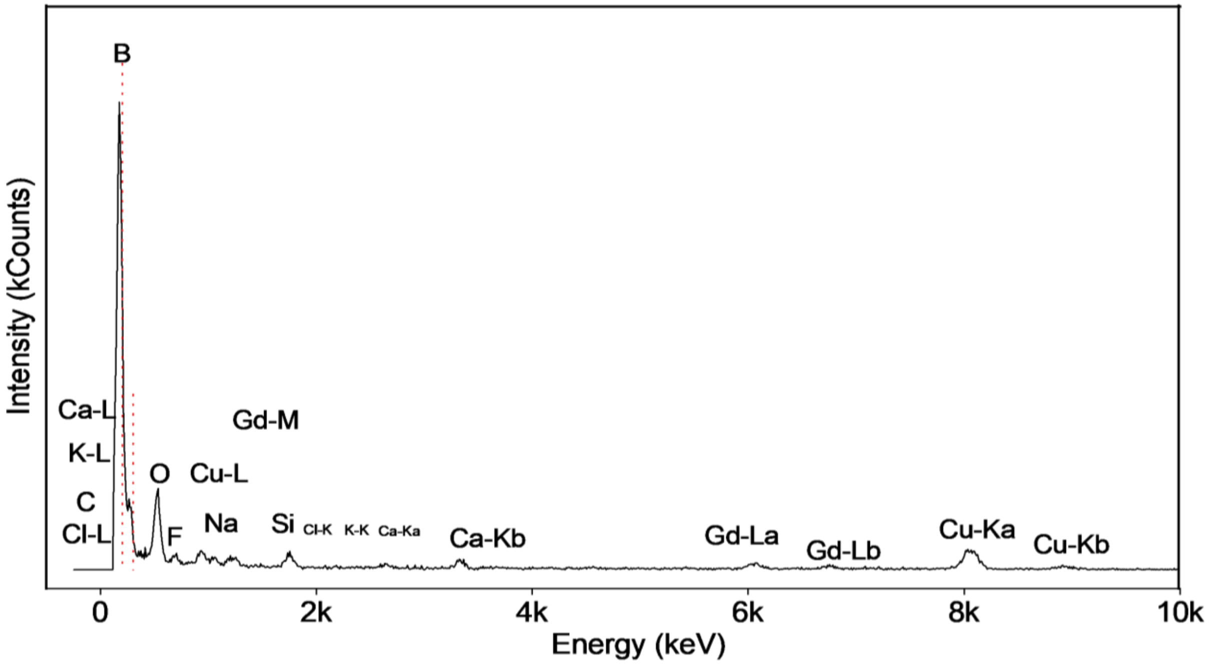

Figure S4. The EDS spectrum from the TEM analysis of the exfoliated borophene nanosheets.

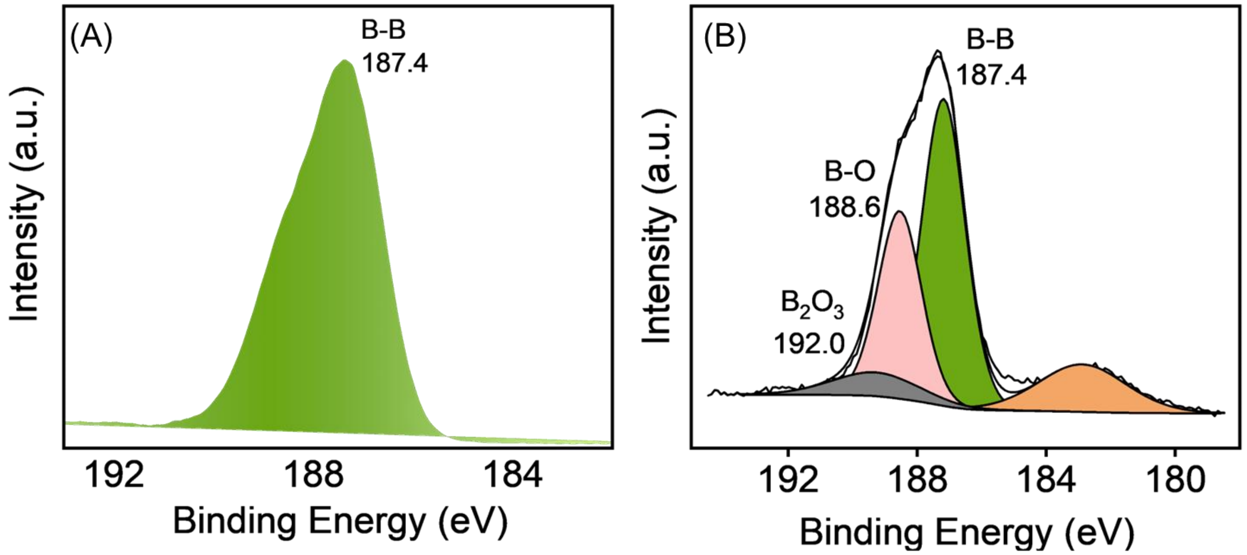

Figure S5. Deconvoluted XPS spectra for (**A**) pristine boron powder and (**B**) synthesized borophene nanosheets.

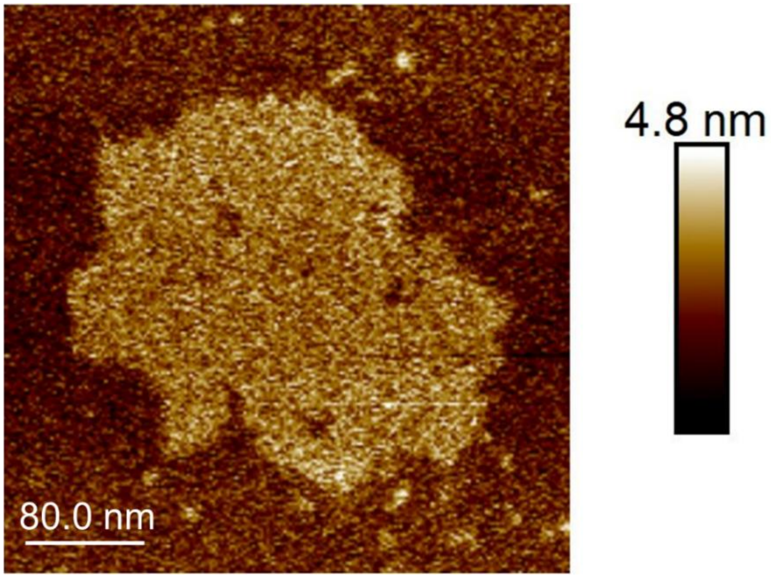

Figure S6. Atomic Force Microscopy (AFM) image with average height H_{avg} =1.10 ± 0.60 nm.

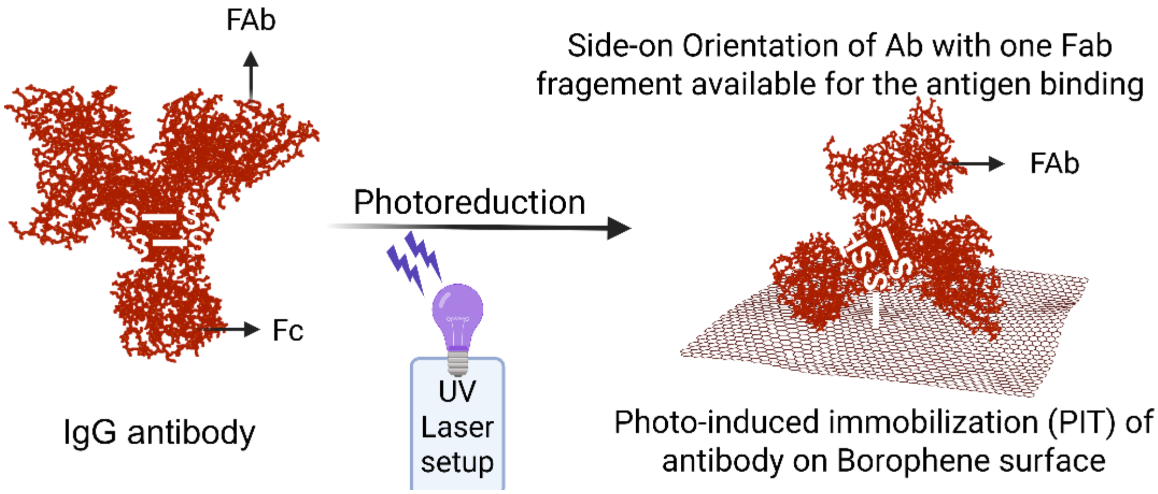

Figure S7. Schematic representation of antibody orientation on borophene through active thiol generated by UV-irradiation.

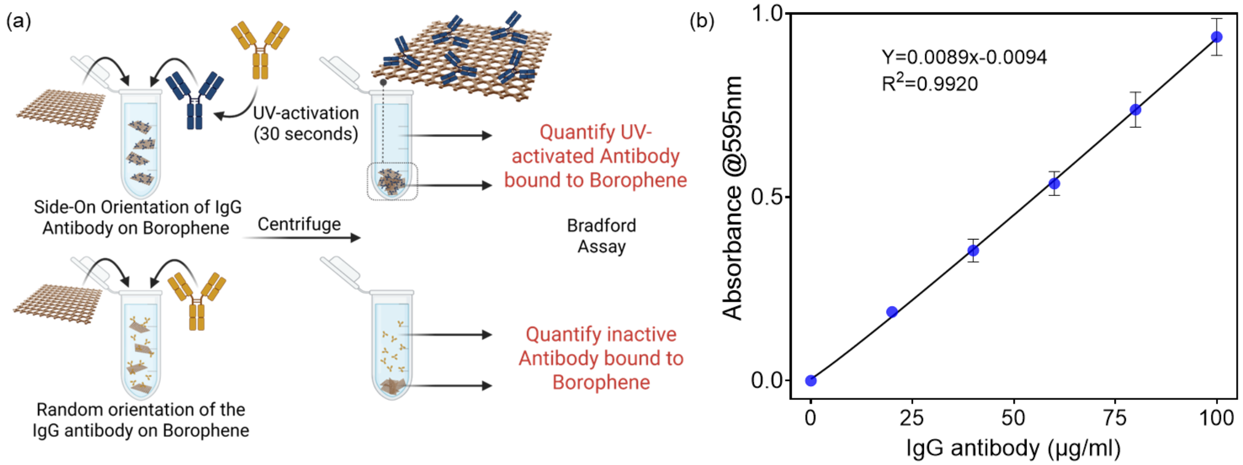

Figure S8. (**a**) Schematic representation of only the antibody and UV-treated (free thiol) antibody conjugation to borophene nanosheet. (**b**) Standard curve for Bradford assay with free antibody.

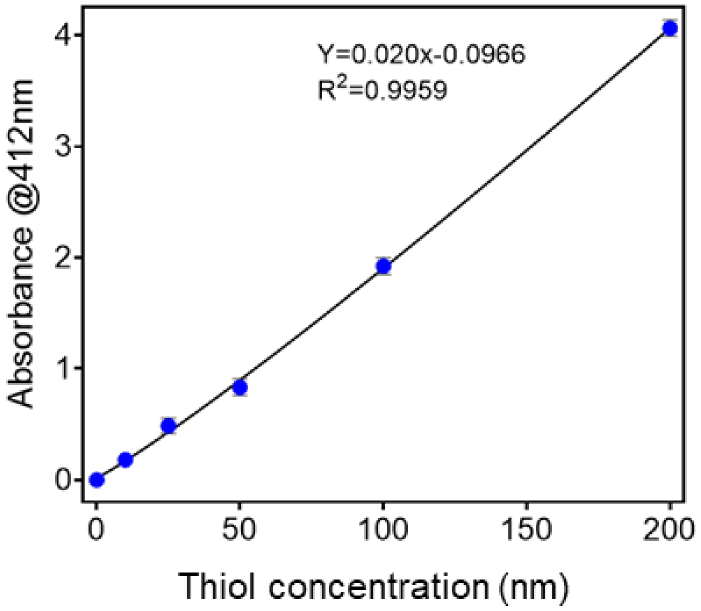

Figure S9. Standard curve obtained with 5,5-dithio-bis (2-nitrobenzoic acid) (DTNB) for free thiol.

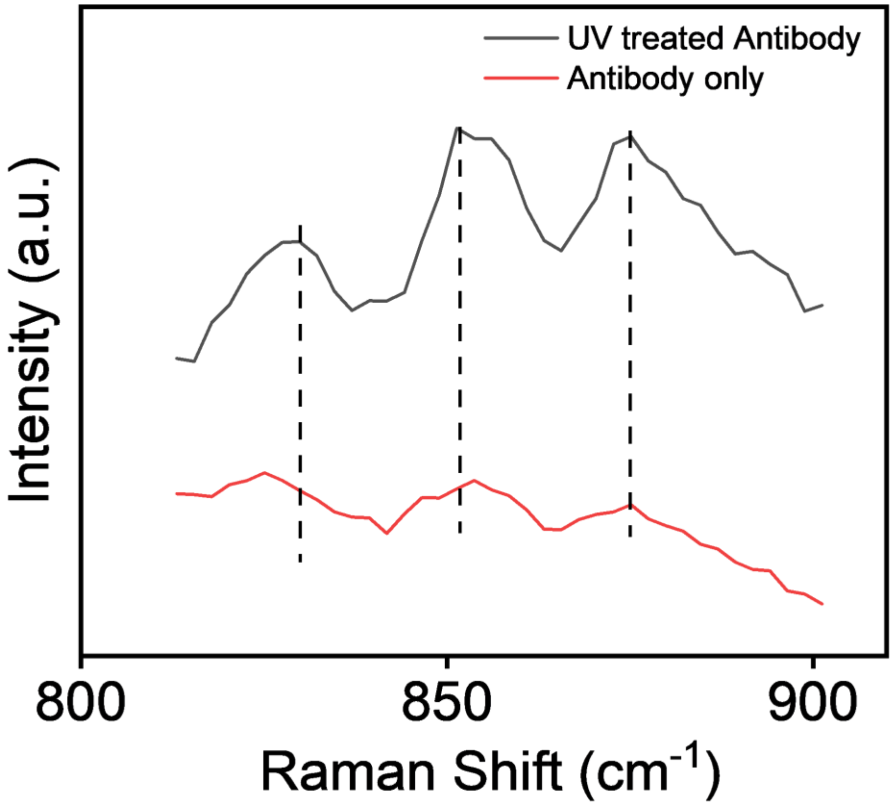

Figure S10. Raman spectral analysis reveals distinct changes in the tyrosine (Tyr) vibrational bands in the UV-treated antibody compared to the untreated control.

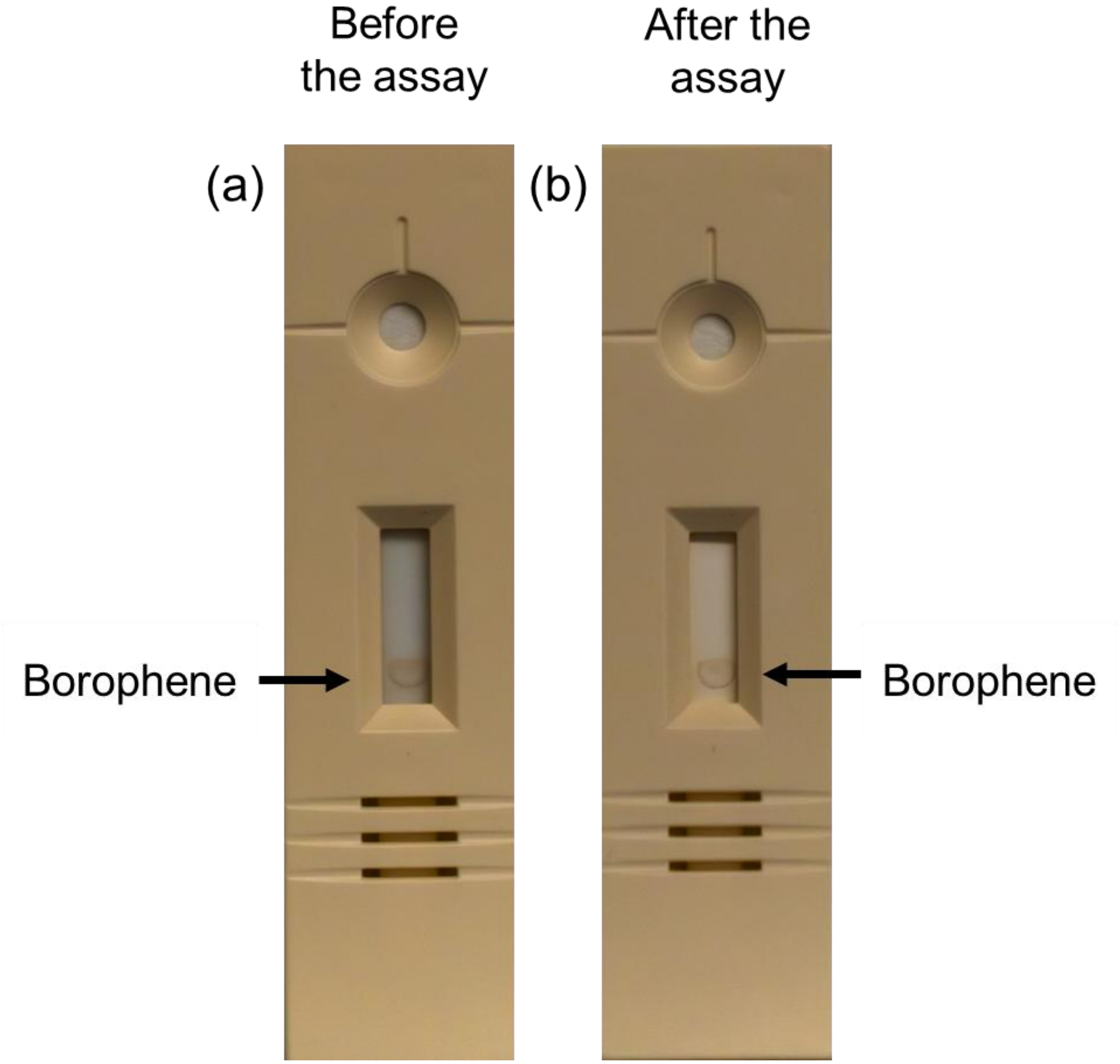

Figure S11. Coating of the nitrocellulose (NC) membrane with borophene: (a) borophene-coated NC membrane prior to the assay; (b) borophene-coated NC membrane following the assay.

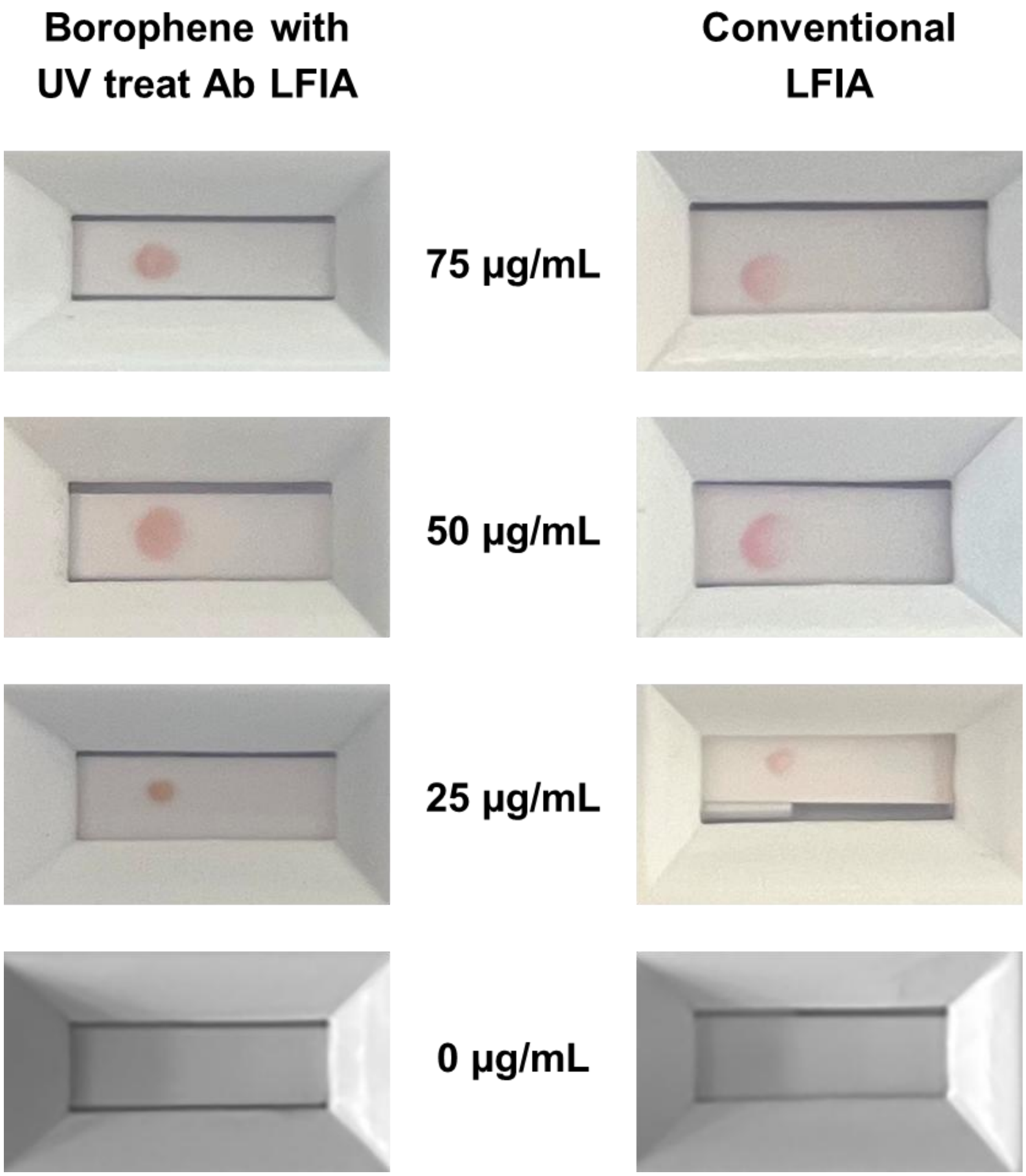

Figure S12 Comparative analysis between traditional gold nanoparticle-based lateral flow immunoassay and borophene-based lateral flow immunoassay strip spot assay

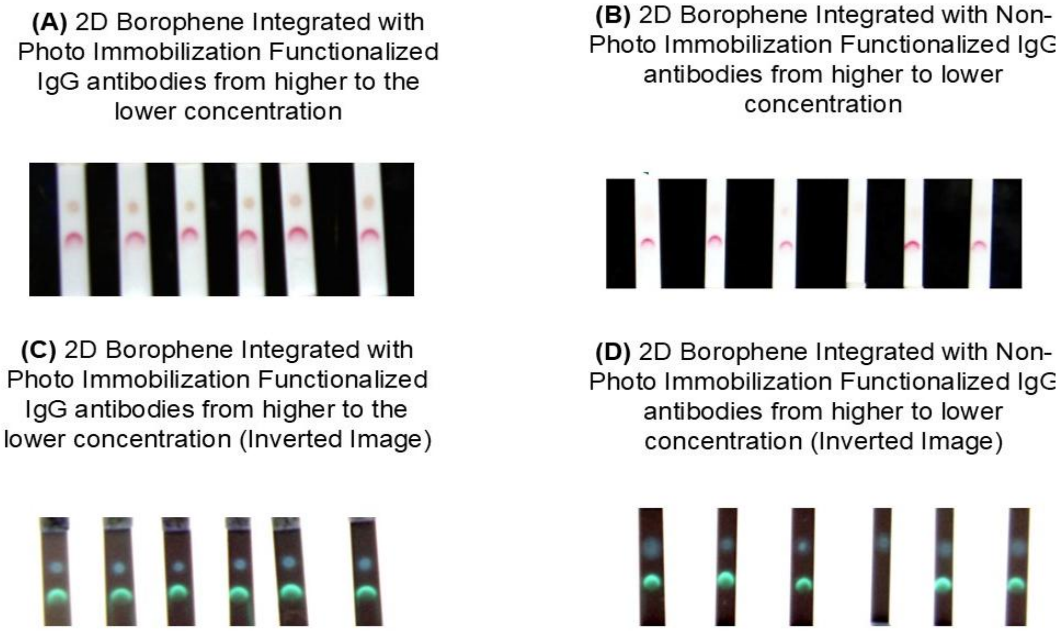

Figure S13. Comparative analysis of the borophene and UV-treated antibody-based lateral flow immunoassay and borophene and UV-untreated antibody-based lateral flow immunoassay. (**a**) Borophene and UV-treated antibody-based lateral flow immunoassay at concentrations (1-6) (**b**) Borophene and UV-untreated antibody-based lateral flow immunoassay at concentrations **(c)** Inverted image of borophene and UV-treated antibody-based LFIA **(d)** Inverted image of borophene and UV-untreated antibody-based LFIA

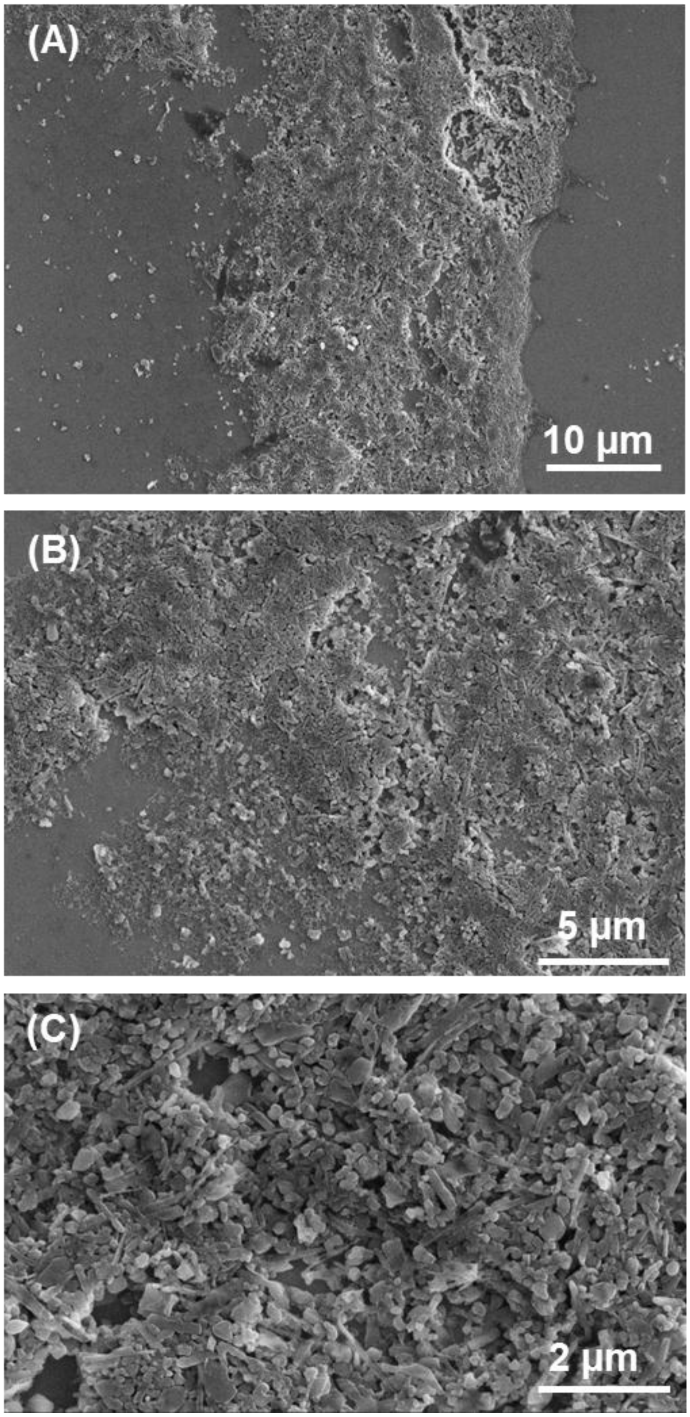

Figure S14. Scanning electron microscopy (SEM) analysis of borophene nanoparticles at (**a**) 7000 × (**b**) 15,000 × and (**c**) 40,000 × magnification. A borophene suspension was deposited over a silica gel and imaged by SEM.

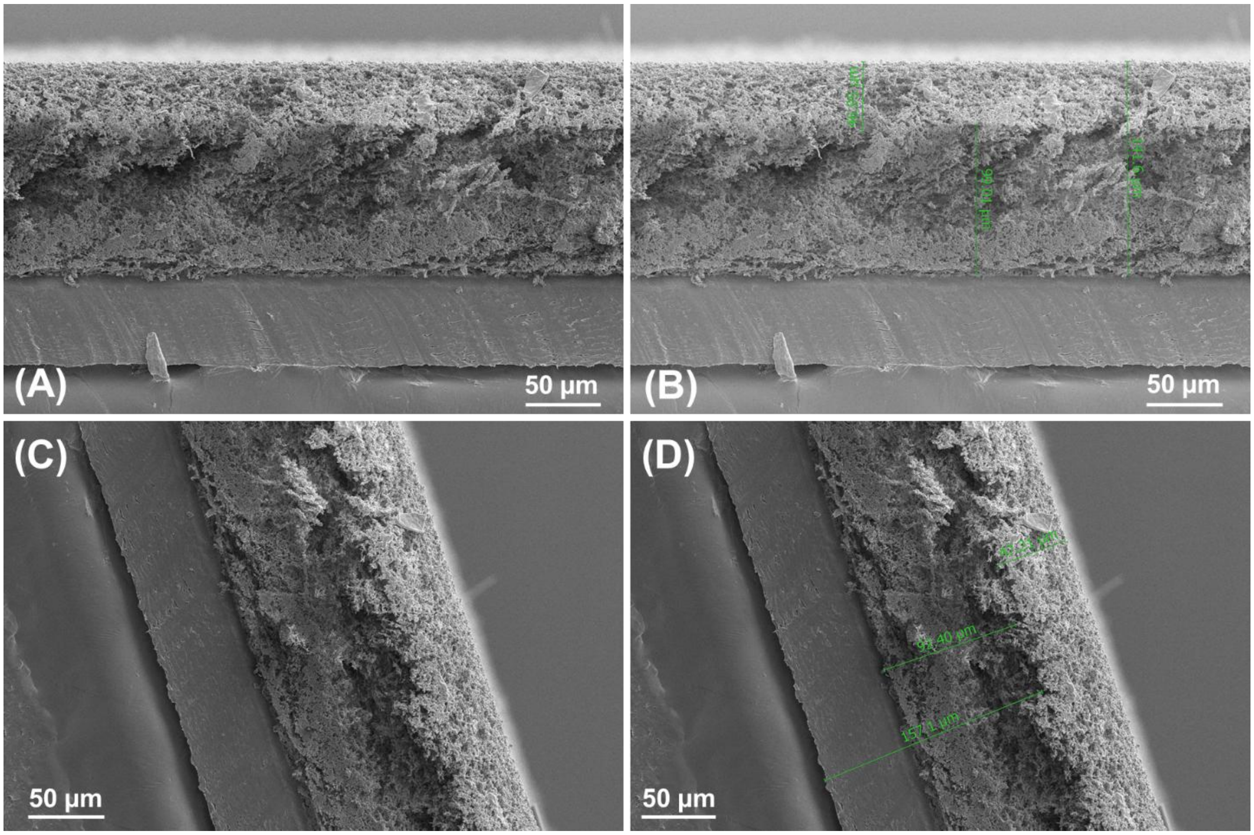

Figure S15. Cross-section analysis of the nitrocellulose NC membrane using scanning electron microscopy (SEM). (**a**) Analysis of the cross-section of the NC+ borophene zone (zone B) at 250× (a) and 1000× (b) magnification. The borophene was incorporated over the NC membrane. The top NC layers penetrated by borophene particles are distinguishable from the bottom NC layers by their lighter color.

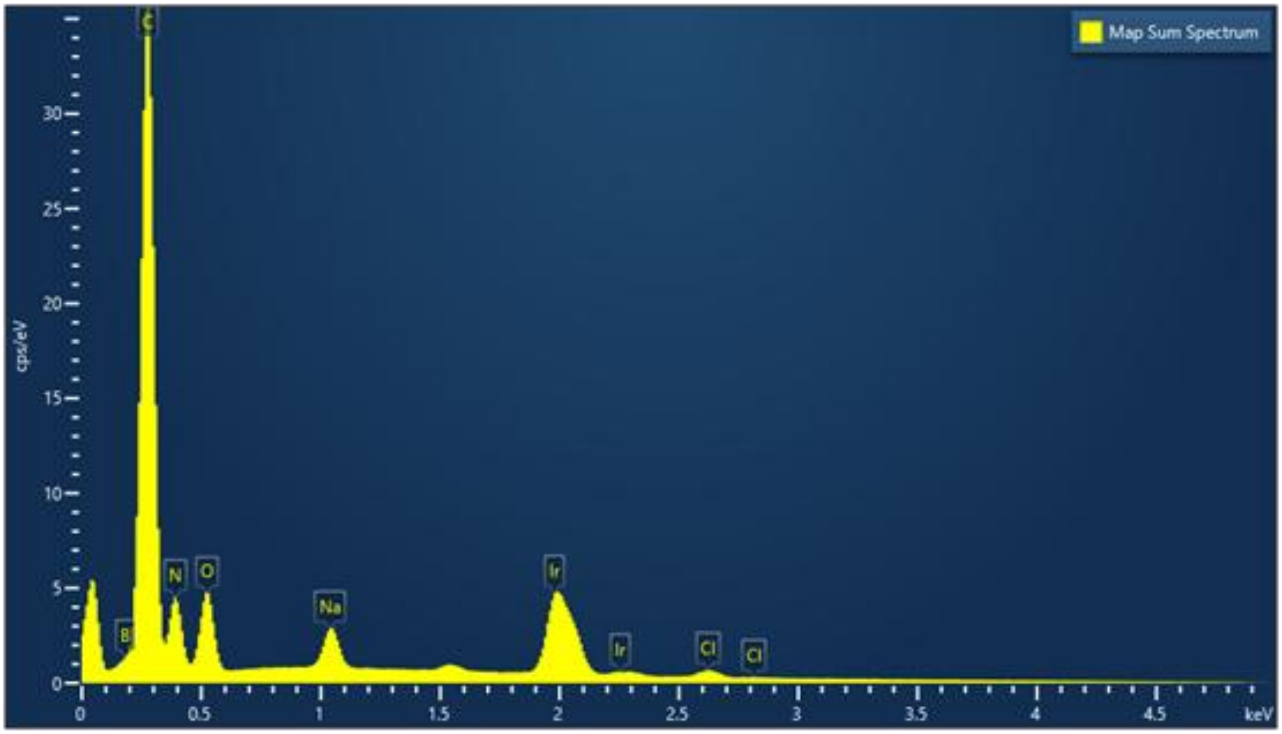

Figure S16. The EDS spectra from the SEM analysis of the borophene incorporated into a nitrocellulose membrane.

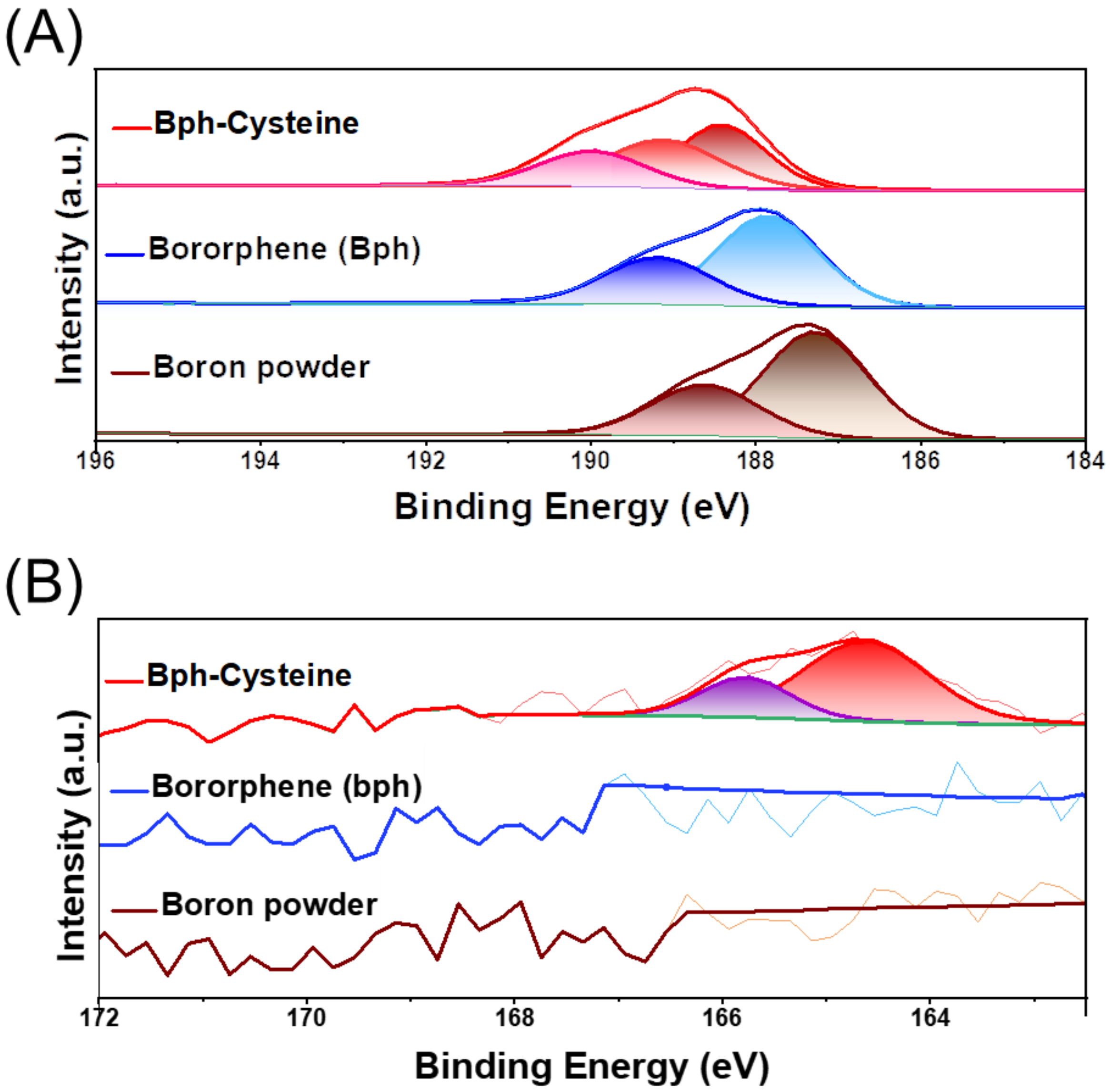

Figure S17. XPS data (**a**) borophene nanosheets and (**b**) cysteine conjugated borophene nanosheets.

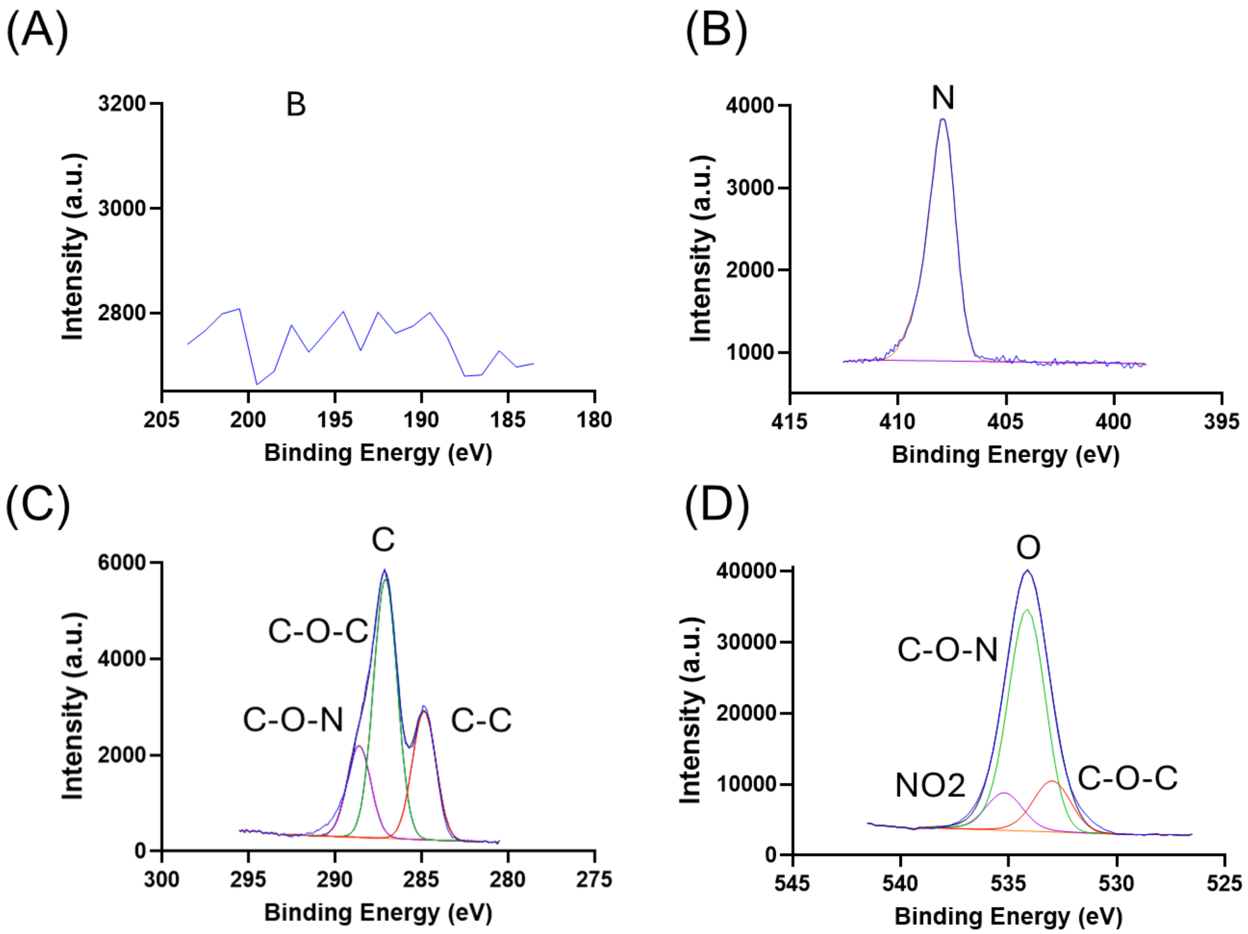

Figure S18. Deconvoluted XPS spectra showing the (**a**) Boron peaks in B1s (**b**) N1s (**c**) C1s (**d**) O1s from the plain nitrocellulose membrane.

Table S1. The relative abundance of the elements identified from XPS (represented in percentage)

Sample	B	C	F	N	O	Si	R-S	SO_3
Borophene and UV-irradiated anti-human HMGB-1 IgG antibody	11.1	59.6	3.2	7.7	17.9	0.7	0.2	0.2

Table S2. Concentration of elements and sulfur species (in atom%)

Samples	B	C	F	N	O	Si	R-S	SO3
Antibody Only	-	42.3		7.8	14		0.3	
Borophene sandwich	11.1	59.6	3.2	7.7	17.9	0.7	0.2	0.2
Tryptophan, $C^{11}H^{12}N^{2}O_2$, expected*	-	73.3	-	13.3	13.3	-	-	-
Cysteine, $C^{3}H^{7}NO_2S$ expected*	-	42.9	-	14.3	28.6	-	14.3	-

Table S3: B-S bond Peak positions in our work and their justification by the Peer-reviewed references.

S. No	Sample	B-S Bond (eV)	References
1	UV irradiated anti-Human HMGB-1 IgG antibody	163.3	Present Study
2	X-Ray Photoelectron B2S3	163.3	[1]
3	Boron and sulfur co-doped graphene	163.3	[2]
4	Basicity of Sulfide Electrolytes Investigated by 11B Solid	163.3	[3]
5	Sulfur-doped graphene quantum dot	163.3	[4]

Table S4. EDS data as acquired from the SEM analysis

Map Sum Spectrum								
Element	Signal Type	Line	Apparent Concentration	k Ratio	Wt%	Wt% Sigma	Atomic %	Standard Name
B	EDS	K series	9.14	0.09145	29.72	1.14	33.45	Pure Element
C	EDS	K series	10.30	0.10305	54.35	0.89	55.07	Pure Element
N	EDS	K series	1.64	0.01134	6.90	0.15	6.00	BN
O	EDS	K series	1.34	0.01174	4.88	0.09	3.71	SiO2
Na	EDS	K series	0.79	0.00673	1.86	0.04	0.99	Albite
Cl	EDS	K series	0.86	0.00824	2.29	0.08	0.79	NaCl
Total					100.00		100.00	

REFERENCES

(1) Gonbeau, D.; Bouih, H.; Pfister-Guillouzo, G.; Menetrier, M.; Levasseur, A. X-ray photoelectron spectrum of glassy B2S3. Experimental and theoretical study. *Journal of the Chemical Society, Faraday Transactions* **1995**, *91* (1), 93-97.
(2) Chatterjee, M.; Nath, P.; Kadian, S.; Kumar, A.; Kumar, V.; Roy, P.; Manik, G.; Satapathi, S. Highly sensitive and selective detection of dopamine with boron and sulfur co-doped graphene quantum dots. *Scientific Reports* **2022**, *12* (1), 9061.
(3) Jiang, S.-K.; Yang, S.-C.; Nikodimos, Y.; Huang, S.-J.; Lin, K.-Y.; Kuo, Y.-H.; Tsai, B.-Y.; Li, J.-N.; Lin, S. D.; Jiang, J.-C. Lewis acid probe for basicity of sulfide electrolytes investigated by 11B solid-state NMR. *JACS Au* **2023**, *3* (8), 2174-2182.
(4) Li, F.; Sun, L.; Luo, Y.; Li, M.; Xu, Y.; Hu, G.; Li, X.; Wang, L. Effect of thiophene S on the enhanced ORR electrocatalytic performance of sulfur-doped graphene quantum dot/reduced graphene oxide nanocomposites. *Rsc Advances* **2018**, *8* (35), 19635-19641.
